# Supplementary figures and images for: Fear memory recall involves hippocampal somatostatin interneurons
Source: PLoS Biol. 2023 Jun 8;21(6):e3002154. doi: 10.1371/journal.pbio.3002154 (PMC10284381; doi:10.1371/journal.pbio.3002154)

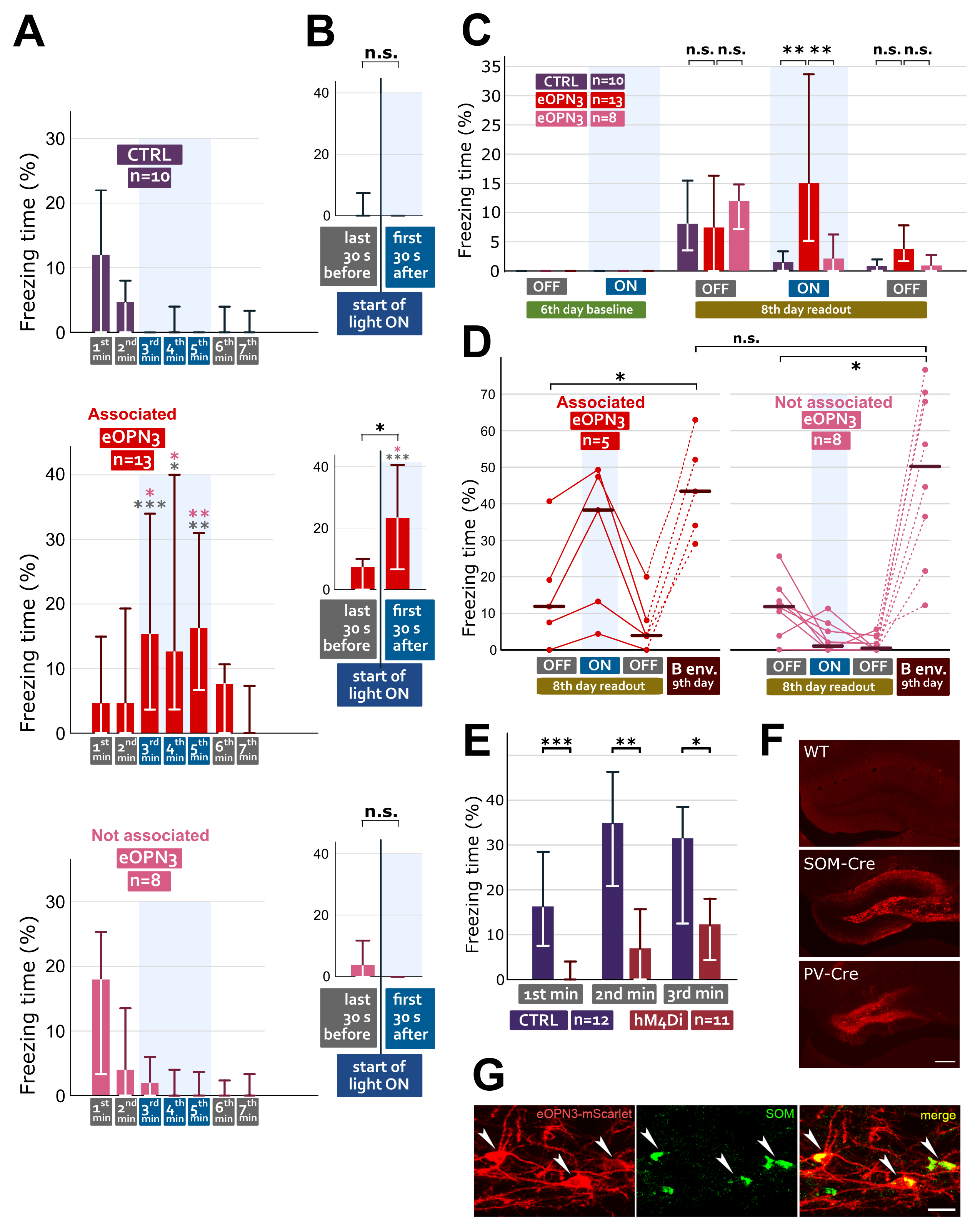

Supplement: S1 Fig — (A) Three graphs show freezing behavior (% of total time) in each minute of the readout sessions (on day 8, in environment “C”) for each mouse in experiments demonstrated in Fig 1, A to D: upper panel CTRL-mice, middle panel Associated eOPN3-mice, lower panel Not-associated eOPN3-mice. Data for CTRL-mice (n = 10, median [25%–75% quartiles]): first min: 12.00 [0.00–22.00], second min: 4.67 [0.00–8.00], third min: 0.00 [0.00–0.00], fourth min: 0.00 [0.00–4.00], fifth min: 0.00 [0.00–0.00], sixth min: 0.00 [0.00–4.00], seventh min: 0.00 [0.00–3.33]. Data for Associated eOPN3-mice (n = 13, median [25%–75% quartiles]): first min: 4.67 [0.00–15.00], second min: 4.67 [0.00–19.33], third min: 15.33 [3.67–34.00], fourth min: 12.67 [3.67–40.00], fifth min: 16.33 [6.67–31.00], sixth min: 7.67 [0.00–10.67], seventh min: 0.00 [0.00–7.33]. Data for Not associated eOPN3-mice (n = 8, median [25%–75% quartiles]): first min: 18.00 [3.33–25.33], second min: 4.00 [0.00–13.50], third min: 2.00 [0.00–6.00], fourth min: 0.00 [0.00–4.00], fifth min: 0.00 [0.00–3.67], sixth min.: 0.00 [0.00–2.33], seventh min: 0.00 [0.00–3.33]. Between-group statistics are labeled on graphs for Associated eOPN3-mice (red): comparison of the third minute period between CTRL and Associated eOPN3-mice: gray ***: p = 0.0003, between Associated eOPN3 and Not-associated eOPN3-mice: pink *: p = 0.027; comparison of the fourth minute period between CTRL and Associated eOPN3-mice: gray *: p = 0.012, between Associated eOPN3 and Not-associated eOPN3-mice: pink *: p = 0.019; comparison of the fifth minute period between CTRL and Associated eOPN3-mice: gray **: p = 0.006, Associated eOPN3 and Not-associated eOPN3-mice: pink **: p = 0.004 (Mann–Whitney U-tests). (B) Three graphs show freezing behavior (% of total time) in 30 s right before and right after the start of the light illumination. Data for CTRL-mice (n = 10, median [25%–75% quartiles]): last 30 s before: 0.00 [0.00–7.33], first 30 s after: 0.00 [0.00–0.00]. S [file pbio.3002154.s001.tif]

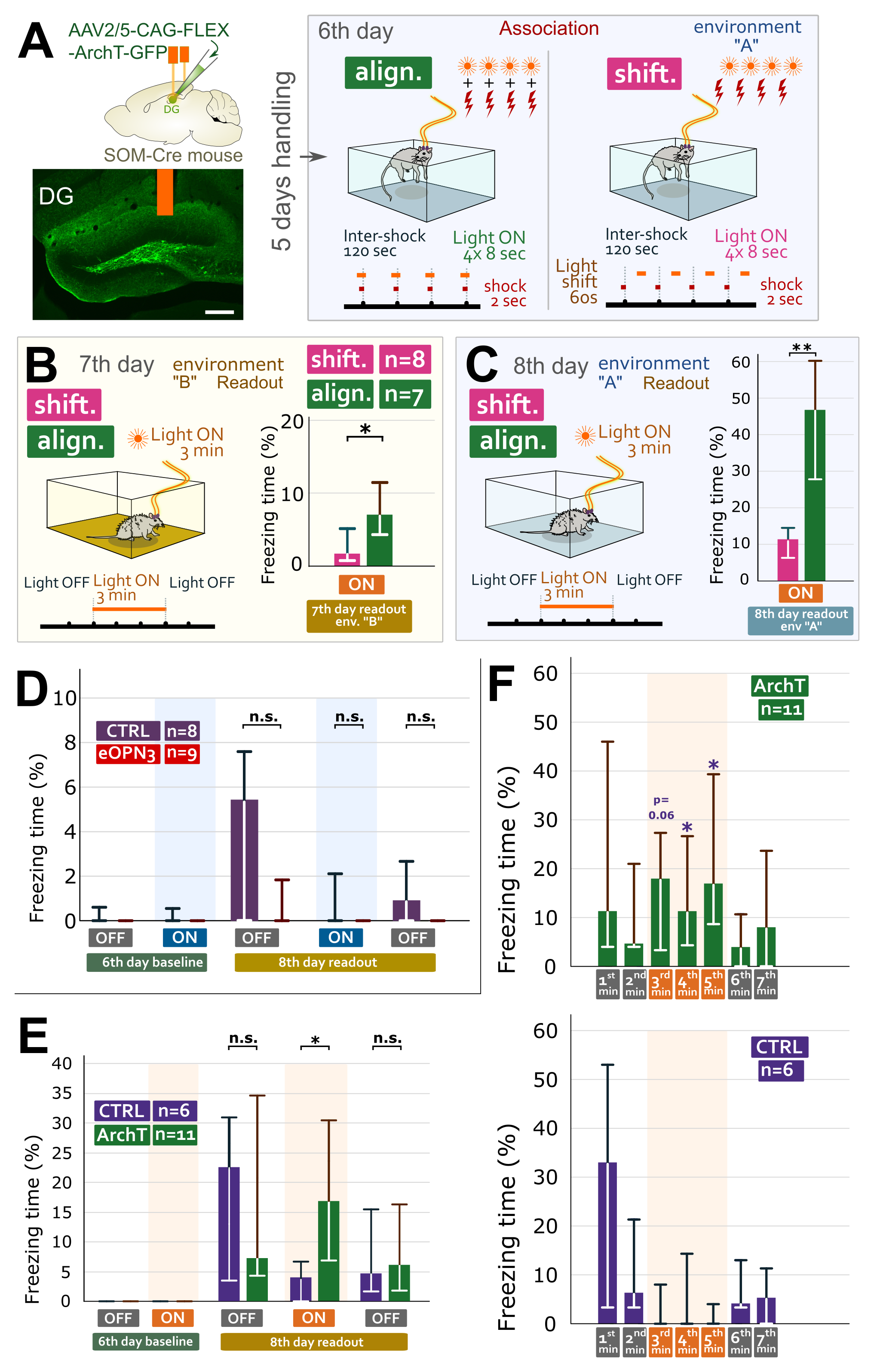

Supplement: S2 Fig — (A) After infecting DG SOM cells with inhibitory ArchT opsin-containing AAVs bilaterally, we implanted optic fibers over DGs. Representative image: injection site and optic fiber position (yellow). Scale bar: 200 μm. Day 6, in environment “A,” “Align. mice” received foot shocks aligned with light illumination, while “Shift. mice” received light illumination 60 s (±2 s) after each foot shocks. (B) Day 7, in environment “B”: 2 min OFF—3 min ON—2 min OFF light cycle. “Align. mice” (dark green) could recall fear memory significantly more efficiently. These ArchT mediated recalls in environment B are significant but less effective than those mediated by eOPN3 that had a longer deactivation time during fear conditioning (Fig 1). The graph shows freezing time during the 3 min light ON period on day 7 in environment “B” (medians and interquartile ranges). Data for “Shift. mice” (n = 8, median [25%–75% quartiles]): readout ON: 2.62 [0.63–5.16]. Data for “Align. mice” (n = 7, median [25%–75% quartiles]): readout ON: 6.90 [4.18–11.39]. Between-group statistics: comparison of the readout first OFF periods between Shift. and Align. mice: n.s.: non-significant, p = 0.954; comparison of the readout ON periods between Shift. and Align. mice: *: p = 0.024; comparison of the readout second OFF periods between Shift. and Align. mice: n.s.: non-significant, p = 0.321 (Mann–Whitney U-tests). (C) Day 8, in environment “A”: 2 min OFF - 3min ON—2 min OFF light cycle. Light illumination is significantly more efficient in “Align. mice.” Graph shows freezing time during 3 min light ON period on day 8 in environment “A” (medians and interquartile ranges). Data for “Shift. mice” (n = 8, median [25%–75% quartiles]): readout ON: 11.02 [6.26–14.52]. Data for “Align. mice” (n = 7, median [25%–75% quartiles]): readout ON: 46.77 [27.80–60.19]. Between-group statistics: comparison of the readout first OFF periods between Shift. and Align. mice: n.s.: non-significant, p = 0.148; comparison of the reado [file pbio.3002154.s002.tif]

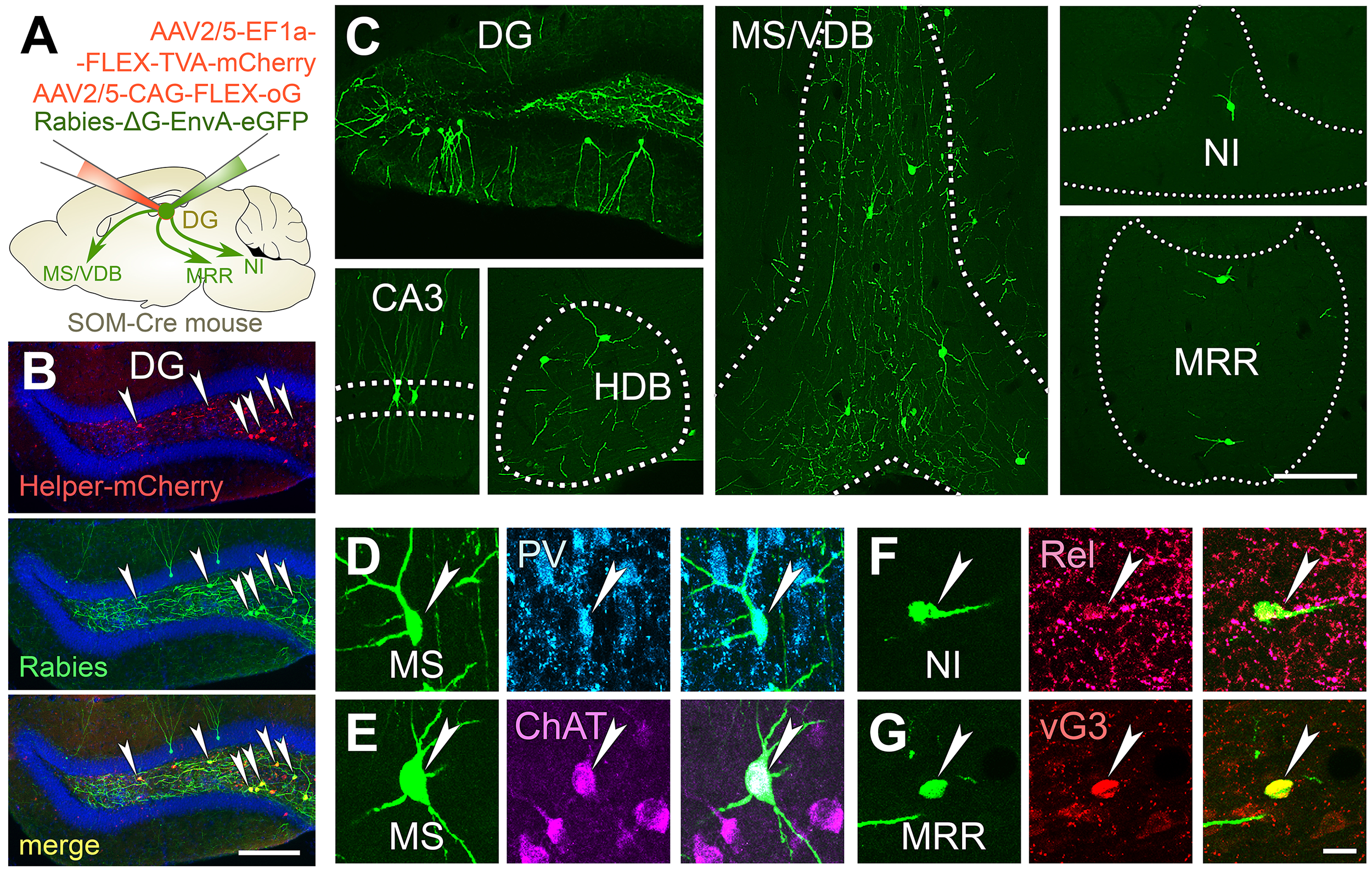

Supplement: S3 Fig — (A) Cre-dependent helper viruses were injected into the dorsal and ventral DG bilaterally of SOM-Cre mice, followed by an injection of Rabies(ΔG)-EnvA-eGFP 5 weeks later (n = 2 mice). (B) Injection site of helper (red) and rabies (green) viruses in the DG. White arrowheads show the starter cells (that express both viruses) in the hilus. Scale bar: 200 μm. (C) Rabies-labeled neurons in different intrahippocampal as well as subcortical brain areas establish synapses on DG SOM-positive neurons. Scale bar: 200 μm. HDB: horizontal diagonal band of Broca, MS/VDB: medial septum/vertical diagonal band of Broca, NI: nucleus incertus, MRR: median raphe region. (D–G) Fluorescent images show that DG SOM cells innervating rabies infected input neurons were clearly positive for PV (6/45) in MS/VDB (D), or positive for ChAT (30/45) in MS/VDB (E), or positive for Relaxin-3 (3/3) in NI (F), or positive for vGluT3 (7/9) in MRR (G). Scale bar: 20 μm for every images. (TIF) [file pbio.3002154.s003.tif]

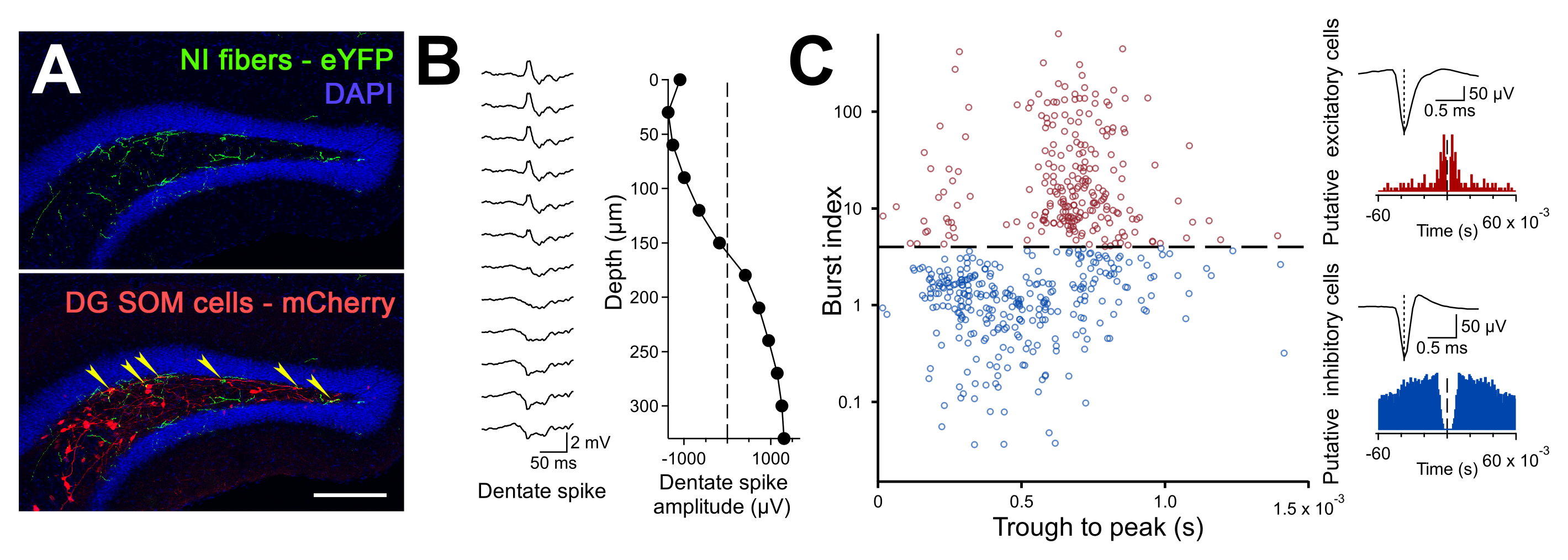

Supplement: S4 Fig — (A) Confocal laser scanning microscopic images from virally injected SOM-Cre/vGAT-Flp mouse (described in Fig 3A) show the location of NI GABAergic fibers (green) in the DG. Arrowheads show putative synaptic contacts of NI fibers onto DG SOM cells (red). Scale bar: 200 μm. (B) Local field potential recordings of a dentate spike at different depth in the hippocampal formation. Right: amplitude of the dentate spike at different depths. Note the reversal of the amplitude. (C) Single units are plotted based on their trough to peak interval and burst indices (the latter is on a logarithmic scale). Dashed horizontal line indicate the separation between the putative excitatory (red) and putative inhibitory units (blue). Putative inhibitory neurons were defined if the burst index was less than 4, while putative excitatory neurons were defined if the burst index was more than 4 (72). Right insets depict sample average waveforms of a putative excitatory (top) and inhibitory units (bottom) from the DG with the corresponding auto-correlograms below them. The data underlying this figure can be found in S1 Data. (TIF) [file pbio.3002154.s004.tif]

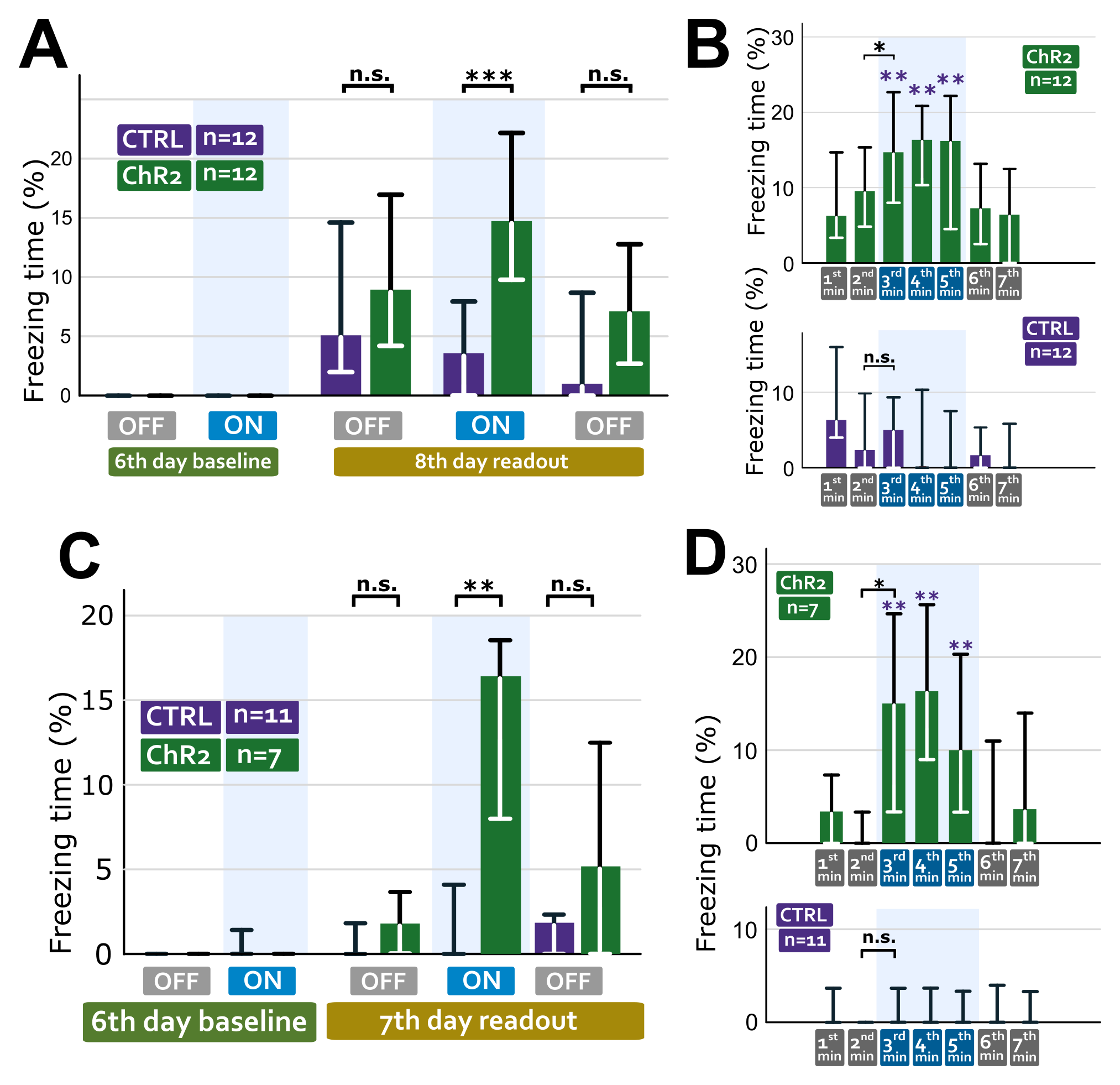

Supplement: S5 Fig — (A) Graph shows freezing time during the 3 min baseline light OFF and 5 min light ON periods on day 6, and freezing time during 2-3-2 min light OFF, ON and OFF periods on day 8, respectively. Data for CTRL-mice (n = 12, median [25%–75% quartiles]): baseline OFF: 0.00 [0.00–0.00], baseline ON: 0.00 [0.00–0.00], readout OFF: 5.08 [2.00–14.58], readout ON: 3.56 [0.00–7.94], readout OFF: 1.00 [0.00–8.67]. Data for ChR2 mice (n = 12, median [25%–75% quartiles]): baseline OFF: 0.00 [0.00–0.00], baseline ON: 0.00 [0.00–0.00], readout OFF: 8.92 [4.17–16.92], readout ON: 14.72 [9.78–22.17], readout OFF: 7.08 [2.67–12.75]. Between-group statistics: comparison of the readout first OFF periods between CTRL and ChR2 mice: n.s.: non-significant, p = 0.488; comparison of the readout ON periods between CTRL and ChR2 mice: ***: p = 0.0007; comparison of the readout second OFF periods between CTRL and ChR2 mice: n.s.: non-significant, p = 0.112 (Mann–Whitney U-tests). (B) Graphs show freezing levels (% of total time) in each minute during the readout session (also in panel A) for CTRL (n = 12) and ChR2 (n = 12) mice. Data for CTRL-mice (n = 12, median [25%–75% quartiles]): first min: 6.33 [4.00–16.00], second min: 2.33 [0.00–9.83], third min: 5.00 [0.00–9.33], fourth min: 0.00 [0.00–10.33], fifth min: 0.00 [0.00–7.50], sixth min: 1.67 [0.00–5.33], seventh min: 0.00 [0.00–5.83]. Statistics: comparison of the third min and fourth min: n.s.: non-significant, p = 0.575 (Wilcoxon signed-rank test). Data for ChR2 mice (n = 12, median [25%–75% quartiles]): first min: 6.17 [3.33–14.67], second min: 9.50 [4.83–15.33], third min: 14.67 [8.00–22.67], fourth min: 16.33 [10.33–20.83], fifth min: 16.17 [4.50–22.17], sixth min: 7.17 [2.50–13.17], seventh min: 6.33 [0.00–12.50]. Statistics: comparison of the second min and third min: black *: p = 0.041 (Wilcoxon signed-rank test). Between-group statistics: comparison of the third minute period between CTRL and ChR2 mice: purple **: p = 0.009; compar [file pbio.3002154.s005.tif]

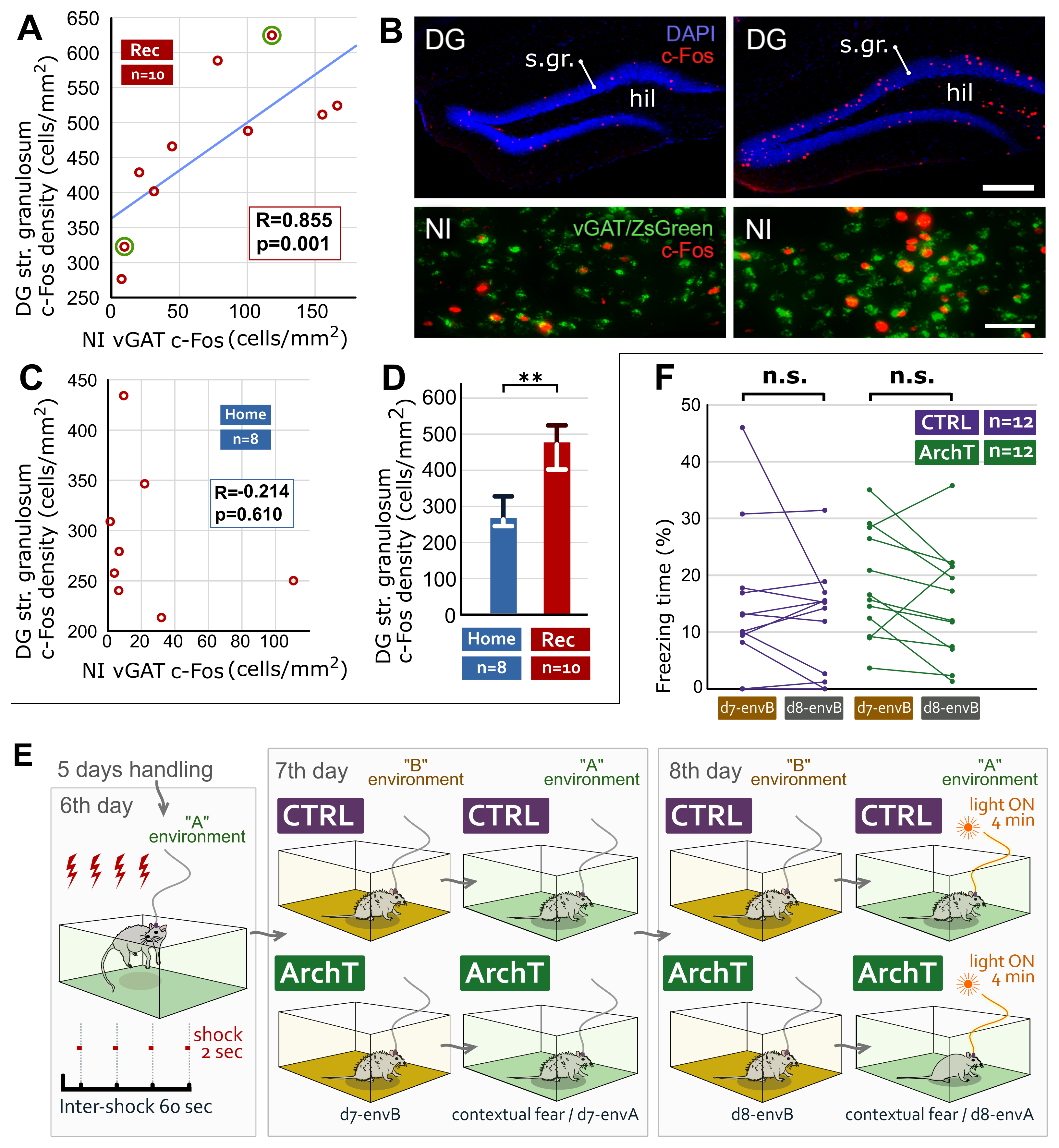

Supplement: S6 Fig — (A, B) Scatterplot shows a significant correlation (Spearman-rank correlation) between the density of c-Fos labeled vGAT positive cells (cells/mm2) in NI and the density of c-Fos labeled cells (cells/mm2) in DG granule cell layer in 10 Recent mice. Representative fluorescent images show c-Fos positive cells (red) in the DG and in the NI from 2 mice the data point of which are labeled with a green circle around the original data point in panel A. These fluorescent images illustrate that the more c-Fos positive GABAergic (green) cells were observed in the NI (lower panel), the more c-Fos positive cells were observed in the granule cell layer of the DG (upper panel) in 10 Recent mice that successfully recalled their memory. Scale bars: 200 μm for DG images, 50 μm for NI images. s.gr.: stratum granulosum, hil: hilus. (C) Scatterplot shows no correlation between the density of c-Fos labeled vGAT positive cells (cells/mm2) in NI and the density of c-Fos labeled cells (cells/mm2) in DG granule cell layer in 8 control “Homecage mice.” Correlation details are as follows: R = −0.214, non-significant: p = 0.610 (Spearman-rank correlation). (D) Differences in density (cells/mm2) of c-Fos positive cells in DG GC layer in Homecage mice and Recent mice (medians and interquartile ranges). Data for Homecage mice (n = 8, median [25%–75% quartiles]): 268.44 [245.24–327.79]. Data for Recent mice (n = 10, median [25%–75% quartiles]): 477.13 [401.98–524.43]. Statistics: **: p = 0.005 (Mann–Whitney U-test). (E) The whole behavioral paradigm of the inhibition of NI cells during contextual memory recall. This illustration explains how fear behavior was tested in a control environment “B” (Fig 5). (F) Graph shows individual data points of the time spent with freezing behavior in environment “B” for each mouse (median [25%–75% quartiles]) on day 7 (d7-envB) and day 8 (d8-envB), respectively (also see panel D). Data for CTRL-mice in d7-envB: 11.56 [8.83–17.33], in d8-envB: 14.72 [1.94–16.28] a [file pbio.3002154.s006.tif]

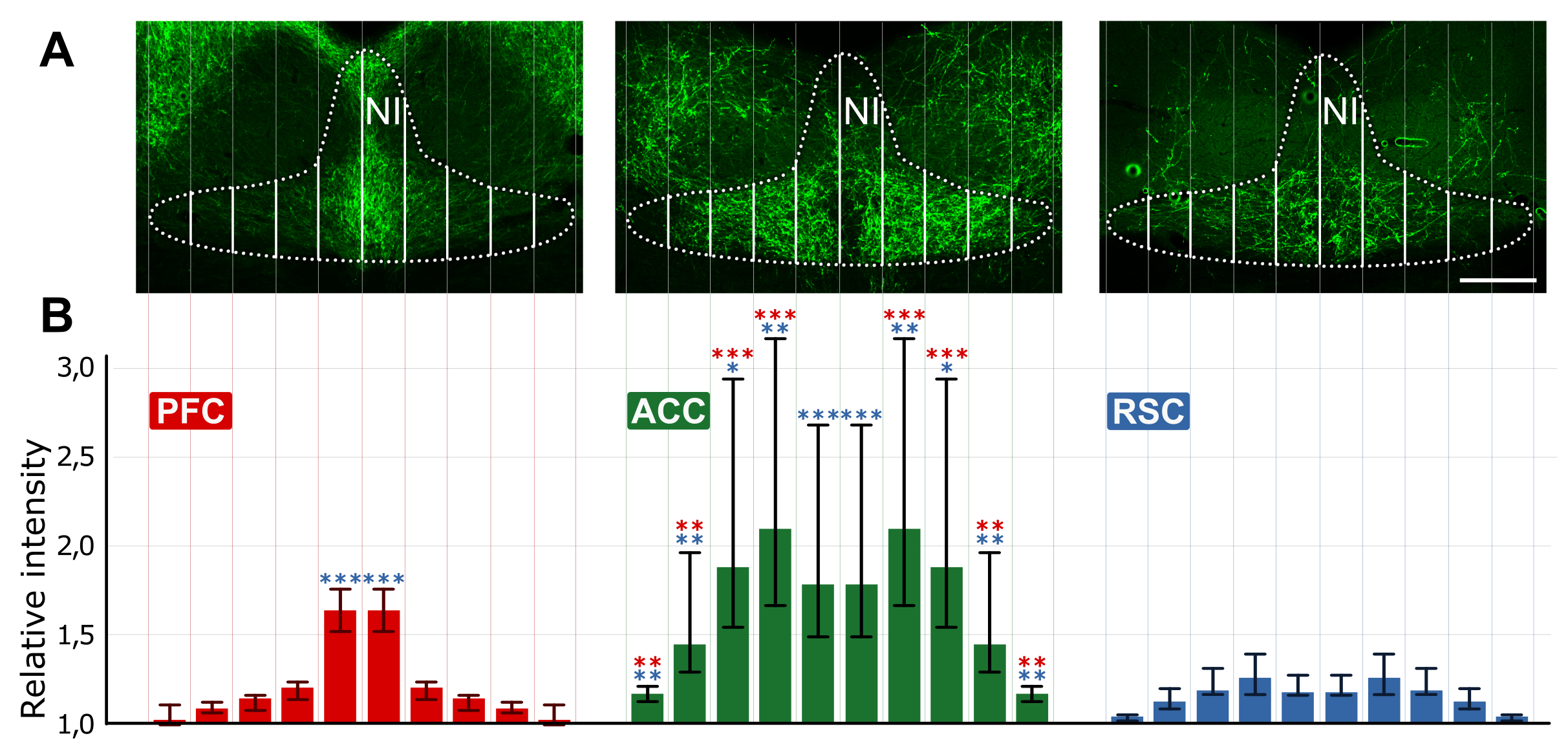

Supplement: S7 Fig — (A) Representative fluorescent images show that NI is innervated by cortical areas differently and illustrates how NI areas were equally divided into 10 sub-areas for measurement. Scale bar: 200 μm. (B) Three graphs show relative pixel intensities for each sub-areas of NI innervated by the PFC, ACC, and RSC, respectively (medians and interquartile ranges). Statistics: 2 mice per group with 12 (PFC) or 10 (ACC, RSC) individual data points per sub-area.*: p < 0.05, **p < 0.01, ***: p < 0.001 (multiple comparisons with Bonferroni-corrected p-values). Blue stars indicate significance between PFC and RSC groups or ACC and RSC groups, red stars indicate significance between ACC and PFC groups. The data underlying this figure can be found in S1 Data. (TIF) [file pbio.3002154.s007.tif]

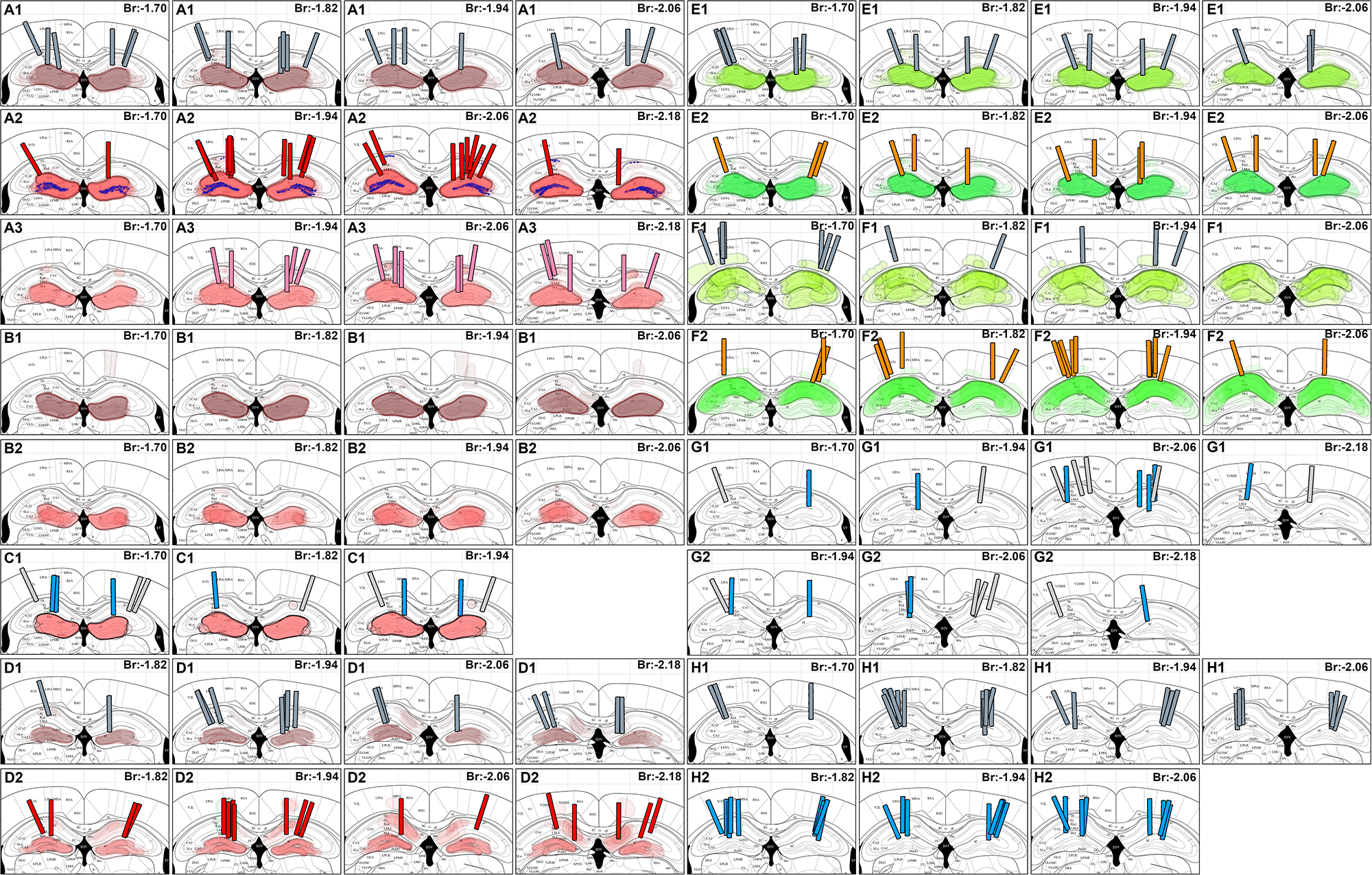

Supplement: S8 Fig — Summary of virus injection sites in the HIPP in every mouse used in the behavioral opto- and chemogenetic experiments. The virus injection sites in different mice from all experiments were analyzed. We localized the exact regions were traces of the virus injection in each section of the region could be detected. Then, we illustrated these areas in the stereological atlas (Paxinos G, Franklin K. 2008. Mouse brain in stereotaxic coordinates). Then, we overlaid these illustrations on each other to get a representative localization of the region of the viral infection around the tip of the optic fiber or around the middle of the injection sites. (A1-3) Images show AAV2/2-EF1α-DIO-mCherry (A1, brown) and AAV2/1-hSyn-SIO-eOPN3-mScarlet (A2-3, pink) virus injection sites and positions of the optic fibers (gray, red, and pink fibers) for mice used in DG SOM cells re-inhibition experiment (described in Fig 1A). Images represent the 10 injection sites of CTRL-mice (A1), 13 injection sites of Associated eOPN3-mice (A2, blue dots in the hilus represent somata of the illuminated cells), and 8 injection sites of Not associated eOPN3-mice (A3). (B1-2) Images show AAV2/2-EF1α-DIO-mCherry (B1, brown) and AAV2/8-hSyn-DIO-hM4D(Gi)-mCherry (B2, pink) virus injection sites for mice used in chemogenetic experiment with DG SOM cells (described in Fig 1H). Images represent the 12 injection sites of CTRL-mice (B1) and 11 injection sites of hM4Di-mice (B2). (C1) Images show AAV2/1-hSyn-SIO-eOPN3-mScarlet (pink) virus injection sites and positions of the optic fibers for eOPN3-mice (n = 6) used in optogenetic c-Fos staining experiment with DG SOM cells (described in Fig 1E). Blue fibers represent the illuminated sides; gray fibers represent the non-illuminated sides. (D1-2) Images show AAV2/2-EF1α-DIO-mCherry (D1, brown) and AAV2/1-hSyn-SIO-eOPN3-mScarlet (D2, pink) virus injection sites and positions of the optic fibers (gray and red fibers) for mice used in DG PV cells re-inhibition experim [file pbio.3002154.s008.tif]

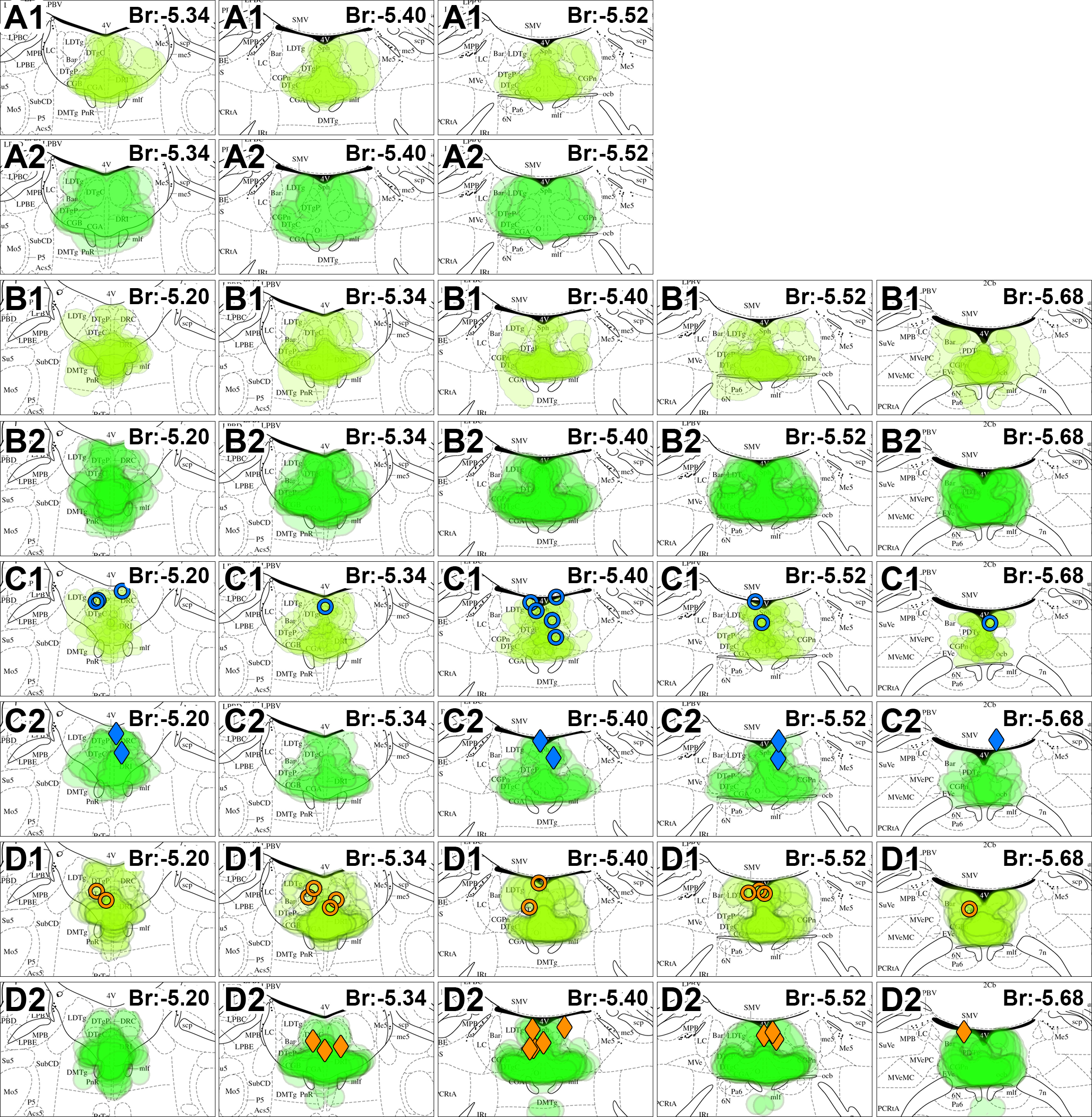

Supplement: S9 Fig — Summary of virus injection sites in the NI in every mouse used in the behavioral optogenetic experiments. The virus injection sites in different mice from all experiments were analyzed. We localized the exact regions where traces of the virus injection in each section of the region could be detected. Then, we illustrated these areas in the stereological atlas (Paxinos G, Franklin K. 2008. Mouse brain in stereotaxic coordinates). Then, we overlaid these illustrations on each other to get a representative localization of the region of the viral infection around the tip of the optic fibers or around the middle of the injection sites. The positions of the tips are represented by the lowest points of the geometric forms. (A1-2) Images show AAV2/5-EF1α-DIO-eYFP (A1, light green) and AAV2/5-EF1α-DIO-ChR2-eYFP (A2, green) virus injection sites for mice used in optogenetic c-Fos experiment of NI DG-projecting fibers (described in Fig 3C). Images represent the 8 injection sites of CTRL-mice (A1) and 5 injection sites of ChR2 mice (A2). (B1-2) Images show AAV2/5-EF1α-DIO-eYFP (B1, light green) and AAV2/5-EF1α-DIO-ChR2-eYFP (B2, green) virus injection sites for mice used in NI DG-projecting fibers re-stimulation experiment (described in Fig 3F). Images represent the 12 injection sites of CTRL-mice (B1) and 12 injection sites of ChR2 mice (B2). (C1-2) Images show AAV2/5-EF1α-DIO-eYFP (C1, light green) and AAV2/5-EF1α-DIO-ChR2-eYFP (C2, green) virus injection sites and positions of the tip of the optic fibers (blue circles, blue diamonds) for mice used in NI cells re-stimulation experiment (described in Fig 4A). Images represent the 11 injection sites of CTRL-mice (C1) and 7 injection sites of ChR2 mice (C2). (D1-2) Images show AAV2/5-EF1α-DIO-eYFP (D1, light green) and AAV2/5-CAG-FLEX-ArchT-GFP (D2, green) virus injection sites and positions of the tip of the optic fibers (orange circles, orange diamonds) for mice used in NI cell inhibition experiment (described in Fig 5G). Imag [file pbio.3002154.s009.tif]

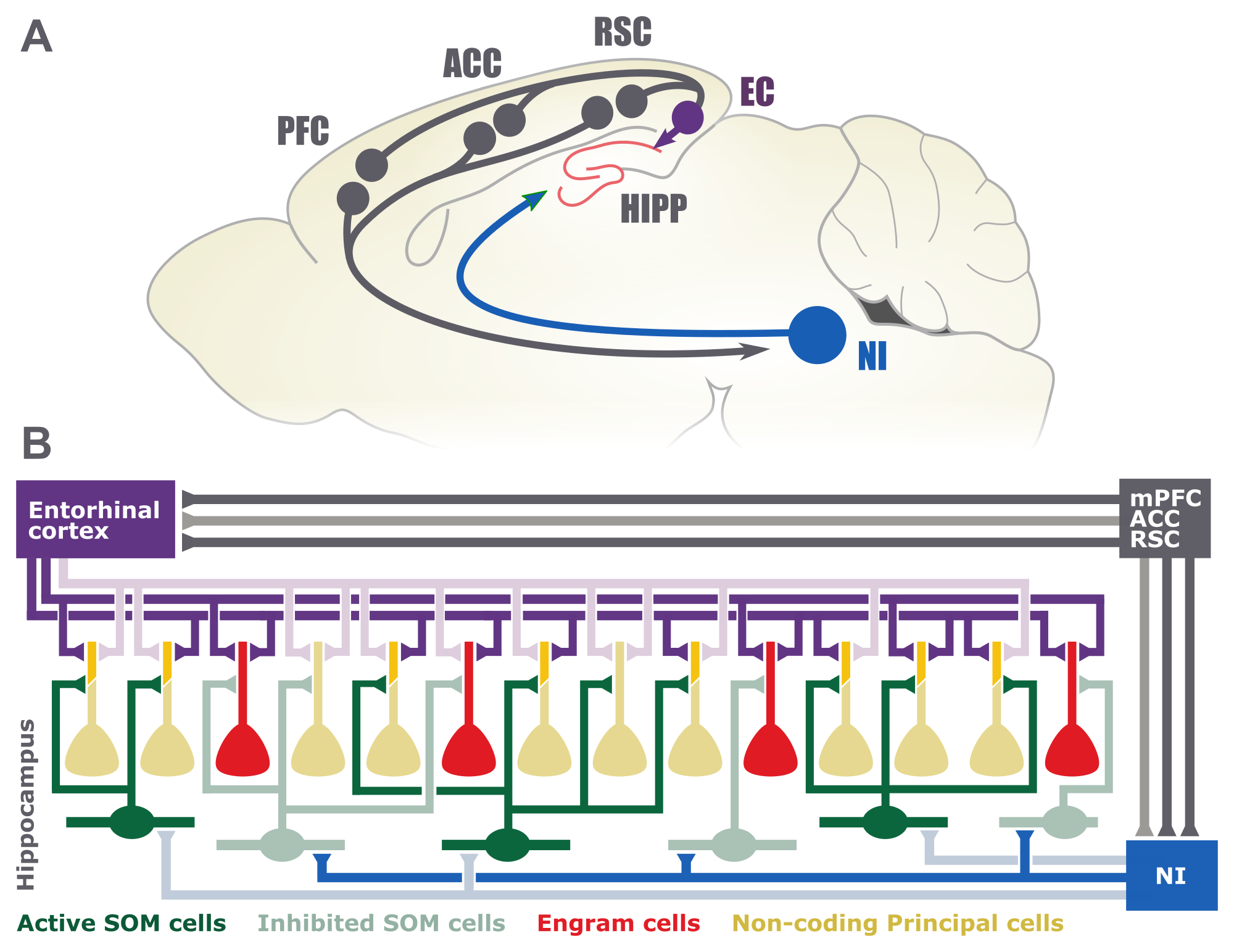

Supplement: S10 Fig — The hippocampus receives contextual fear processing-related excitatory inputs from cortical areas partly via the entorhinal cortex (A, B, purple) that broadcasts a rough, only partly context-specific activation pattern to the dendrites of principal neurons (15). This gives some principal cells only the opportunity to become a memory trace-encoding engram cell, because most principal cells are kept inhibited by dendrite-targeting SOM cells, whereas others do not even get strong enough inputs. On the other hand, the hippocampus receives inhibitory inputs from the brainstem nucleus incertus (NI) as well that initiates fear memory-specific disinhibition of principal neurons via local hippocampal SOM cells (B, green). This gives an overlapping population of principal cells permission to become a memory trace-encoding engram cell (red). This mechanism may be initiated by memory-processing neocortical centers (mPFC, ACC, RSC) via both the entorhinal cortex and the NI simultaneously. The interaction of these (and other modulatory) inputs may provide the basic mechanism for both the formation and reactivation of engram cells in the hippocampus region. (Dark colors illustrate active and light colors illustrate inactive fibers, cells or dendritic compartments.). (TIF) [file pbio.3002154.s010.tif]
