# Supplementary material for: Fear memory recall involves hippocampal somatostatin interneurons
Source: PLoS Biol. 2023 Jun 8;21(6):e3002154. doi: 10.1371/journal.pbio.3002154 (PMC10284381; doi:10.1371/journal.pbio.3002154)
Supplement: S1 Extended Data — (DOCX) [file pbio.3002154.s015.docx]

Extended Data for Main Figure 1:

**Figure 1B:** Graph shows individual percentages of time spent with freezing behavior during the light OFF-ON-OFF cycles for each mouse on day 8. Freezing behavior data are given in % of freezing time of total time spent in environment “C” during light OFF, ON and OFF periods.

Data for CTRL-mice (n=10, median [25%-75% quartiles]): OFF: 8.17 [3.50-15.50], ON: 1.50 [0.00-3.33], OFF: 0.83 [0.00-2.00]. Statistics: comparison of OFF to ON period: *: p=0.028; ON to OFF period: n.s.: non-significant, p=0.600 (Wilcoxon signed-rank tests).

Data for Associated eOPN3-mice (n=13, median [25%-75% quartiles]): OFF: 7.50 [0.00-16.33], ON: 15.00 [5.11-33.67], OFF: 3.83 [1.67-7.83]. Statistics: comparison of OFF to ON period: **: p=0.006; ON to OFF period: ***: p=0.0009, (Wilcoxon signed-rank tests).

Data for Not-associated eOPN3-mice (n=8, median [25%-75% quartiles]): OFF: 12.00 [7.17-14.83], ON: 2.11 [0.00-6.22], OFF: 0.83 [0.00-2.75]. Statistics: comparison of OFF to ON period: n.s.: non-significant, p=0.063; ON to OFF period: n.s.: non-significant, p=0.173, (Wilcoxon signed-rank tests).

Between-group statistics: comparison of the first OFF periods between CTRL and Associated eOPN3-mice: n.s.: non-significant, p=0.778; between Associated eOPN3-mice and Not-associated eOPN3-mice: n.s.: non-significant, p=0.466 (Mann-Whitney U-tests).

**Figure 1C:** Columns show significant differences in the changes of freezing behavior between the first light OFF and ON periods for each group (median [25%-75% quartiles]) on day 8 in environment “C”.

Data for CTRL-mice: -5.19 [(-11.00)-1.33], n=10. Data for Associated eOPN3-mice: 7.06 [5.11-17.33], n=13. Data for Not-associated eOPN3-mice: -8.44 [(-13.72)-(-2.58)], n=8.

Statistics: comparison of CTRL vs. Associated eOPN3-mice: ***: p=0.0001; comparison of Associated eOPN3-mice vs. Not-associated eOPN3-mice: **: p=0.0016 (Mann-Whitney U-tests).

**Figure 1D:** Graph shows freezing time differences between groups during the light ON period on day 8 in environment “C” (median [25%-75% quartiles]). Data for CTRL-mice: 1.50 [0.00-3.33], n=10. Data for Associated eOPN3-mice: 15.00 [5.11-33.67], n=13. Data for Not-associated eOPN3-mice: 2.11 [0.00-6.22], n=8.

Statistics: comparison of CTRL vs. Associated eOPN3-mice: **: p=0.001; comparison of Associated eOPN3-mice vs. Not-associated eOPN3-mice: **: p=0.007 (Mann-Whitney U-tests).

**Figure 1G:** Graph shows differences in the density of c-Fos positive cells (cells/mm2) in DG granule cell layer in non-illuminated (non) and in light illuminated, that is the disinhibited (inh.) side of the DG (median [25%-75% quartiles]) for n=6 eOPN3-mice.

Data for the non-illuminated (non) side: 257.99 [220.39-452.17]. For the the disinhibited (inh.) side: 409.17 [316.64-470.14]. Statistics: *: p= 0.028, (Wilcoxon signed-rank test).

**Figure1I:** Graph shows that contextual fear of hM4Di-mice almost completely diminished after CNO injection compared to CTRL-mice on day 7 (median [25%-75% quartiles]).

Data for CTRL-mice: 27.00 [17.06-36.00], n=12. Data for hM4Di-mice: 5.22 [3.67-12.89], n=11. Statistics: **: p=0.0025, (Mann-Whitney U-test).

**Figure1J:** About 61% (471/773) of immunolabeled SOM cells were infected with hM4Di-mCherry-containing AAV in the dorsal DG (n=9 hM4Di-mice). Individually these data are the following: mouse1 (53/88), mouse2 (45/73), mouse3 (17/20), mouse4 (83/126), mouse5 (53/77), mouse6 (22/66), mouse7 (75/104), mouse8 (82/121), mouse9 (41/98).
