# Supplementary material for: Fear memory recall involves hippocampal somatostatin interneurons
Source: PLoS Biol. 2023 Jun 8;21(6):e3002154. doi: 10.1371/journal.pbio.3002154 (PMC10284381; doi:10.1371/journal.pbio.3002154)
Supplement: S2 Extended Data — (DOCX) [file pbio.3002154.s016.docx]

Extended Data for Main Figure 2:

**Figure 2B:** Graph shows freezing time differences between PV-Cre mouse groups during the light ON period on day 8 in environment “C” (median [25%-75% quartiles]). Data for CTRL-mice: 0.00 [0.00-2.11], n=8. Data for eOPN3-mice: 0.00 [0.00-0.00], n=9.

Statistics: comparison of CTRL vs. eOPN3-mice: n.s.: non-significant: p=0.219 (Mann-Whitney U-test).

**Figure 2C:** Graph shows individual percentages of time spent with freezing behavior during the light OFF-ON-OFF cycles for each PV-Cre mouse on day 8. Freezing behavior data are given in % of freezing time of total time spent in environment “C” during light OFF, ON and OFF periods.

Data for CTRL-mice (n=8, median [25%-75% quartiles]): OFF: 5.42 [0.00-7.58], ON: 0.00 [0.00-2.11], OFF: 0.92 [0.00-2.67]. Statistics: comparison of OFF to ON period: *: p=0.043; ON to OFF period: n.s.: non-significant, p=0.893 (Wilcoxon signed-rank tests).

Data for eOPN3-mice (n=9, median [25%-75% quartiles]): OFF: 0.00 [0.00-1.83], ON: 0.00 [0.00-0.00], OFF: 0.00 [0.00-0.00]. Statistics: comparison of OFF to ON: n.s.: non-significant, p=0.109; ON to OFF: n.s.: non-significant, p=0.480 (Wilcoxon signed-rank tests).

**Figure 2D:** Columns show no significant difference in the changes of freezing behavior between the first light OFF and ON periods for each PV-Cre mouse group (median [25%-75% quartiles]) on day 8 in environment “C”.

Data for CTRL-mice: -2.53 [(-6.97)-0.00] n=8. Data for eOPN3-mice: 0.00 [(-2.83)-0.00], n=9.

Statistics: comparison of CTRL vs. eOPN3-mice: n.s.: non-significant, p=0.376 (Mann-Whitney U-test).

**Figure 2F:** Graph shows individual percentages of time spent with freezing behavior during the light OFF-ON-OFF cycles for each CA1 SOM-Cre mouse on day 8. Freezing behavior data are given in % of freezing time of total time spent in environment “C” during light OFF, ON and OFF periods.

Data for CTRL-mice (n=6, median [25%-75% quartiles]):]. OFF: 22.67 [3.50-31.00], ON: 4.06 [0.00-6.67], OFF: 4.75 [1.67-15.50]. Statistics: comparison of OFF to ON period: *: p=0.046; ON to OFF period: n.s.: non-significant, p=0.753 (Wilcoxon signed-rank tests).

Data for ArchT-mice (n=11, median [25%-75% quartiles]): OFF: 7.33 [4.33-34.67], ON: 16.89 [6.89-30.44], OFF: 6.17 [1.83-16.33]. Statistics: comparison of OFF to ON: n.s.: non-significant, p=0.351, ON to OFF: *: p=0.016 (Wilcoxon signed-rank tests).

**Figure 2G:** Columns show significant differences in the changes of freezing behavior between the first light OFF and ON periods for each CA1 SOM-Cre mouse group (median [25%-75% quartiles]) on day 8 in environment “C”. Data for CTRL-mice: -14.72 [(-31.00)-(-3.50)], n=6. Data for ArchT-mice: 4.89 [(-4.89)-13.78], n=11. Statistics: comparison of CTRL vs. ArchT-mice: *: p=0.024 (Mann-Whitney U-test).

**Figure 2H:** Graph shows freezing time differences between CA1 SOM-Cre mouse groups during the light ON period on day 8 in environment “C” (median [25%-75% quartiles]). Data for CTRL-mice: 4.06 [0.00-6.67], n=6. Data for ArchT-mice: 16.89 [6.89-30.44], n=11. Statistics: comparison of CTRL vs. ArchT-mice: *: p=0.018 (Mann-Whitney U-test).
