# Supplementary material for: Fear memory recall involves hippocampal somatostatin interneurons
Source: PLoS Biol. 2023 Jun 8;21(6):e3002154. doi: 10.1371/journal.pbio.3002154 (PMC10284381; doi:10.1371/journal.pbio.3002154)
Supplement: S3 Extended Data — (DOCX) [file pbio.3002154.s017.docx]

Extended Data for Main Figure 3:

**Figure 3B:** Our measurements showed that at least 87% (509/583) of virally labeled NI axonal terminals establish gephyrin-labeled synaptic contacts on virally labeled DG SOM cells (n=2 mice). The rest of the target cell may have also been SOM cells without fully labeled dendrites.

**Figure 3E:** Graphs show differences in the density of c-Fos positive cells (cells/mm2) in DG granule cell layer in the non-illuminated and in the illuminated (disinhibited) side of the DG.

Data for the non-illuminated (non) side of CTRL-mice: 346.43 [277.81-375.83]. Data for the illuminated (illu.) side of CTRL-mice: 299.81 [255.35-363.89]. Data for the non-illuminated (non) side of ChR2 mice: 350.12 [285.87-375.48]. Data for the illuminated (illu.) side of ChR2 mice: 425.20 [415.12-433.86].

Statistics: Paired comparisons (non vs. illu.) in CTRL-mice: n.s.: non-significant, p=0.263, in ChR2 mice: *: p=0.043 (Wilcoxon signed-rank tests).

Between-group statistics: CTRL non vs. ChR2 non: n.s.: non-significant, p=0.942; CTRL illu. vs. ChR2 illu.: *: p=0.048, (Mann-Whitney U-tests).
