# Supplementary material for: Fear memory recall involves hippocampal somatostatin interneurons
Source: PLoS Biol. 2023 Jun 8;21(6):e3002154. doi: 10.1371/journal.pbio.3002154 (PMC10284381; doi:10.1371/journal.pbio.3002154)
Supplement: S4 Extended Data — (DOCX) [file pbio.3002154.s018.docx]

Extended Data for Main Figure 4:

**Figure 4B:** Graph shows individual percentages of time spent with freezing behavior during the light OFF-ON-OFF cycles for each mouse on day 8. Freezing behavior data are given in % of freezing time of total time spent in environment “B” during light OFF, ON and OFF periods.

Data for CTRL-mice (n=12, median [25%-75% quartiles]): OFF: 5.08 [2.00-14.58], ON: 3.56 [0.00-7.94], OFF: 1.00 [0.00-8.67]. Statistics: comparison of OFF to ON period: **: p=0.007; ON to OFF period: n.s.: non-significant, p=0.779 (Wilcoxon signed-rank tests).

Data for ChR2 mice (n=12, median [25%-75% quartiles]): OFF: 8.92 [4.17-16.92], ON: 14.72 [9.78-22.17], OFF: 7.08 [2.67-12.75]. Statistics: comparison of OFF to ON period: *: p=0.012; ON to OFF period: *: p=0.023, (Wilcoxon signed-rank tests).

Between-group statistics: comparison of the first OFF period between CTRL and ChR2 mice: n.s.: non-significant, p=0,488 (Mann-Whitney U-test).

**Figure 4C:** Graph shows freezing time differences between groups during the light ON period on day 8 in environment “C” (median [25%-75% quartiles]).

Data for CTRL-mice: 3.56 [0.00-7.94], n=12. Data for ChR2 mice: 14.72 [9.78-22.17], n=12. Statistics: comparison of CTRL vs. ChR2 mice: ***: p=0.0007 (Mann-Whitney U-test).

**Figure 4D:** Columns show significant differences in the changes of freezing behavior between the first light OFF and ON periods for each group (median [25%-75% quartiles]) on day 8 in environment “B”.

Data for CTRL-mice: -2.44 [(-6.81)-(-0.11)], n=12. Data for ChR2 mice: 4.19 [1.92-7.81], n=12. Statistics: comparison of CTRL vs. ChR2 mice: ***: p=0.0003 (Mann-Whitney U-test).

**Figure 4F:** Graph shows freezing time differences between groups during the light ON period on day 7 in environment “C” (median [25%-75% quartiles]).

Data for CTRL-mice: 0.00 [0.00-4.11], n=11. Data for ChR2 mice: 16.44 [8.00-18.56], n=7. Statistics: comparison of CTRL vs. ChR2 mice: **: p=0.001 (Mann-Whitney U-tests).

**Figure 4G:** Graph shows individual percentages of time spent with freezing behavior during the light OFF-ON-OFF cycles for each mouse on day 7. Freezing behavior data are given in % of freezing time of total time spent in environment “C” during light OFF, ON and OFF periods.

Data for CTRL-mice (n=11, median [25%-75% quartiles]): OFF: 0.00 [0.00-1.83], ON: 0.00 [0.00-4.11], OFF: 1.83 [0.00-2.33]. Statistics: comparison of OFF to ON period: n.s.: non-significant: p=0.612; ON to OFF period: n.s.: non-significant, p=0.499 (Wilcoxon signed-rank tests).

Data for ChR2 mice (n=7, median [25%-75% quartiles]): OFF: 1.83 [0.00-3.67], ON: 16.44 [8.00-18.56], OFF: 5.17 [0.00-12.50]. Statistics: comparison of OFF to ON period: *: p=0.018; ON to OFF period: *: p=0.028, (Wilcoxon signed-rank tests).
