# Supplementary material for: Fear memory recall involves hippocampal somatostatin interneurons
Source: PLoS Biol. 2023 Jun 8;21(6):e3002154. doi: 10.1371/journal.pbio.3002154 (PMC10284381; doi:10.1371/journal.pbio.3002154)
Supplement: S5 Extended Data — (DOCX) [file pbio.3002154.s019.docx]

Extended Data for Main Figure 5:

**Figure 5D:** Graphs show differences in the density of c-Fos positive GABAergic cells (cells/mm2) in the NI for Home (n=8), Recent (n=10) and Remote (n=7) mouse groups (median [25%-75% quartiles]).

Data for Home mice: 8.17 [5.25-27.00], for Recent mice: 61.43 [20.50-118.03] and for Remote mice: 87.43 [62.78-138.52].

Statistics: Home vs. Recent mice: *: p=0.029; Home vs. Remote mice: **: p=0.006, Recent vs. Remote mice: n.s.: non-significant: p=0.306 (Mann-Whitney U-tests).

**Figure 5E:** Graph shows contextual fear responses (in % of total time) of Recent mice and Remote mice during the 5 minutes readout period on the 8th and the 37th days, respectively (medians [25%-75% quartiles]).

Data for Recent mice: 18.10 [9.27-19.07], n=10 and for Remote mice: 46.80 [40.53-58.13], n=7.

**Figure 5F:** Scatterplot shows a significant correlation between the density of c-Fos labeled vGAT positive cells (cells/mm2) in NI and the density of c-Fos labeled cells (cells/mm2) in DG granule cell layer in Recent mice (n=10).

Correlation details of the density of c-Fos labeled vGAT positive cells in NI and the density of c-Fos labeled cells in str. granulosum of DG for n=10 Recent mice are as follows: R=0.855, **: p=0.001 (Spearman-rank correlation).

**Figure 5H:** Graph shows individual percentages of time spent with freezing behavior during the light OFF cycles (CFC7) and light ON cycles (CFC8) for each mouse on day 7 and 8, respectively (median [25%-75% quartiles]). CFC: contextual fear conditioning readout day.

Data for CTRL-mice in CFC7: 14.78 [4.44-31.17], in CFC8: 9.56 [4.11-20.00] and n=12. Data for ArchT-mice in CFC7: 21.39 [16.72-29.61], CFC8: 7.61 [4.44-15.33] and n=12.

Statistics: CTRL-mice in CFC7 vs. CFC8: n.s.: non-significant: p=0.158, (Wilcoxon signed-rank test). CTRL-mice in CFC7 vs. ArchT-mice CFC7: n.s.: non-significant: p=0.403, (Mann-Whitney U-test). ArchT-mice in CFC7 vs. CFC8: **: p=0.002 (Wilcoxon signed-rank test).

**Figure 5I:** Columns show significant differences in the changes of freezing behavior (1-CFC8/CFC7) between the light OFF (CFC7) and ON (CFC8) periods for each group (median [25%-75% quartiles]).

Data for CTRL-mice: -27.59 [1.96-(-52.31)]. Data for ArchT-mice: -60.81 [(-44.11)-(-75.59)]. *: p=0.030, (Mann-Whitney U-test).
