# Supplementary material for: Fear memory recall involves hippocampal somatostatin interneurons
Source: PLoS Biol. 2023 Jun 8;21(6):e3002154. doi: 10.1371/journal.pbio.3002154 (PMC10284381; doi:10.1371/journal.pbio.3002154)
Supplement: S6 Extended Data — (DOCX) [file pbio.3002154.s020.docx]

Extended Data for Main Figure 6:

**Figure 6K:** Our measurements showed that at least 79% (34/43) of HIPP-projecting NI cells received altogether 96 (average: 2.82) Homer-1 positive synaptic contacts onto their soma or proximal dendrites from vGluT1-positive mPFC neurons (n=2 mice).

**Figure 6L:** Our measurements showed that at least 84% (57/68) of HIPP-projecting NI cells received altogether 239 (average: 4.19) Homer-1 positive synaptic contacts onto their soma or proximal dendrites from vGluT1-positive ACC neurons (n=3 mice).

**Figure 6M:** Our measurements showed that at least 84% (36/43) of HIPP-projecting NI cells received altogether 114 (average: 3.17) Homer-1 positive synaptic contacts onto their soma or proximal dendrites from vGluT1-positive RSC neurons (n=3 mice).
