# Supplementary material for: Fear memory recall involves hippocampal somatostatin interneurons
Source: PLoS Biol. 2023 Jun 8;21(6):e3002154. doi: 10.1371/journal.pbio.3002154 (PMC10284381; doi:10.1371/journal.pbio.3002154)
Supplement: Attachment — Submitted filename: Zicho_Response to Reviewers.pdf [file pbio.3002154.s021.pdf]

Response to comments by the Reviewers

Research Article for PLOS Biology ID: PBIOLOGY-D-22-01994R1
Title: Fear memory recall via hippocampal somatostatin interneurons

We thank the reviewers for their constructive comments and suggestions and welcome their positive comments on our manuscript. Below we present the Reviewers' unchanged comments in full (in black text), together with our point-by-point responses (in blue text). In the manuscript, we have also indicated in blue text the changes that relate to the Reviewers' suggestions.

Reviewer: 1

Contextual fear memory traces are believed to consist of subsets of hippocampal principal neurons that are specifically activated during the process of memory formation, which couples the CS (conditioned stimulus; a specific context here) with US (unconditioned stimulus; footshock here); moreover, the recall of the specific memory requires the re-activation of the same subset of principal neurons. Previous studies indicate that somatostatin (SOM)-expressing interneurons, which receive excitatory inputs from the medial septum and inhibitory inputs from the nucleus incertus (NI), provide a global inhibitory tone (lateral inhibition) for the selective activation of the memory engram (M. Lovett-Barron et al., 2014; Stefanelli et al., 2016). More recently, Nyiri's group reports that coupling the optogenetic stimulation and inhibition of NI GABAergic neurons with US prevented and facilitated the formation of fear memories, respectively (Szőnyi et al., 2019). Here, Zichó et al. from the same group report that coupling the optogenetic inhibition of hippocampal SOM cells or optogenetic activation of NI GABA neurons during memory formation enables future fear memory recall using the same optogenetic manipulation. To reconcile the seemingly discrepant observations between this study and the Szőnyi et al., 2019 study, the authors hypothesized that inhibiting a subset of hippocampal SOM neurons or activating NI GABA cells may not only disrupt the association between the context and the footshock (as shown in the Szőnyi et al., 2019 study) but also enable the formation of a new memory that associates the activity change of these neurons with the footshock, and the re-inhibition of SOM neurons or re-activation of NI GABA neurons leads to memory recall (Paragraphs 2-3 in Discussion). In essence, the authors seemed to theorize that the optogenetic disturbance of neuronal activity serves as CS here. I feel that the current study provides some novel observations and raises an intriguing interpretation. However, the authors need to provide additional experimental evidence and improve the quality of current experiments to strengthen their conclusions.

We are grateful for Reviewer 1 for her/his constructive and detailed comments and hope that the additional series of experiments suggested by the Reviewer will provide further evidence for our findings.

Major comments:

(1) One can make two predictions based on the authors' hypothesis. First, the timing of inhibiting hippocampal SOM neurons or activating NI GABA cells does not need to be strictly aligned with footshock. Rather, a range of interval duration between the optogenetic manipulations and US should also support memory formation and recall, whereas such manipulations after US shall be ineffective. Data from this new experiment will be critical for establishing authors' key conclusion.

We think that both scenarios would be equally interesting and none of them would alter our main conclusions, however, indeed, the effect of specific timing is an interesting and important question as well. Therefore, at the Reviewer's request, we performed a new series of experiments to demonstrate the effect of the timing of the inhibition of DG SOM cells (see Supplementary Figure S2A-C). Indeed, we found that the associations were significantly more effective when optogenetic inhibition was precisely aligned to the aversive stimulus, rather than presented after a temporal shift (Figure S2A-C). The text and the figures were updated accordingly.

(2) Second, the GABAergic input from the NI is not truly special, in that any other GABAergic input to hippocampal SOM neurons will also support similar fear memory formation and recall. The authors may

address this concern by using cell type-specific rabies tracing to illustrate the presynaptic partners of DG SOM neurons and then test and discuss the potential roles of those inputs. For example, do some other inhibitory inputs also contribute to memory formation and recall, whereas the excitatory inputs lack such effect?

Even if it were not a special input, no study has ever shown similar modulation of fear memories via the hindbrain or the HIPP SOM cells. Nevertheless, most of the inputs to SOM cells originates from the hippocampus and there are no data in the literature about other extra-hippocampal GABAergic inputs, except for the non-specific septal GABAergic innervation that targets several populations of interneurons and is known to primarily contribute to hippocampal rhythmicity. Basal forebrain cholinergic neurons target all hippocampal neurons and contribute to hippocampal rhythmicity and attention as well. Therefore, the extra-hippocampal GABAergic input from the NI to DG SOM cells is known to be quite unique. However, whole-brain analysis of the synaptic input sources of DG SOM cells has not yet been published, probably because even monosynaptic-rabies tracking experiments have limitations with respect to inputs from long-distance nuclei.

Nevertheless, at the Reviewer's request, we have extended these studies ever further. Therefore, in a series of experiments we investigated the brain-wide sources of GABAergic inputs to SOM cells. Using state-of-the-art monosynaptic-rabies technologies, we found that DG SOM cells indeed receive extra-hippocampal GABAergic inputs from medial septal GABAergic cells that are known to facilitate network synchronization and innervate other inhibitory cell types as well in the hippocampus (Salib et al. 2019). DG SOM cells also received medial septum cholinergic inputs, which target different interneurons and all principal cells and play a role in attention. In addition, we found that DG SOM cells received inputs from some non-GABAergic median raphe vGluT3+ cells that target other hippocampal interneuron types as well (Varga et al. 2009). Finally, we found that they receive a very specific and strong input from the relaxin positive NI GABAergic cells that we studied here. We presented these results in Supplementary Figure S3C-G.

Furthermore, although we have previously shown that NI fibers preferentially target HIPP SOM neurons (Szőnyi et al., 2019), at this Reviewer's request, here we also investigated this specifically in the DG in a series of experiments. Now, we found that at least 87% (509/583) of the virally labeled NI axonal terminals formed inhibitory synapse-specific gephyrin-labeled synaptic contacts with virally labeled DG SOM cells. The unidentified targets may also be SOM cells, the dendrites of which were not completely filled with viral tracer protein. This unique target selectivity is presented in the supplementary data section: "Extended Data for Main Fig 3". The text and the figures were updated accordingly.

(3) Several experiments need to be done more rigorously. For example, the authors used c-Fos immunopositivity as indicators of neuron activation in the hippocampus and the NI (Figs. 1 and 5). Given the known limitations of c-Fos labeling, fiber photometry of Ca2+ signals will provide stronger support to the authors claims. In Figs. 3 and 6, the authors used the proximity of FP-labeled NI terminals to hippocampal SOM neurons and NI GABA neurons as indicators of synaptic connections. Slice physiology is required to rigorously establish functional connectivity.

c-Fos labeling is widely used to assess neuronal activation in the literature and this functional connectivity was further confirmed by the significant correlation between the c-Fos activity of NI neurons and DG granule cells. Furthermore, the functional connectivity was also demonstrated by the hippocampus-dependent behavioral effects demonstrated in this study. Nevertheless, the study can indeed further benefit from additional data regarding this connectivity. Unfortunately, the resolution of fiber photometry is insufficient in this case, whereas in vivo physiology seems to be a better choice than slice physiology.

Therefore, we carried out two series of in vivo electrophysiological experiments. Here, we not only demonstrated that NI GABAergic neurons can directly disinhibit and reorganize firing patterns in the DG, but we also proved that the cortical inputs of the NI from the anterior cingulate cortex (ACC) can also reorganize DG firing patterns that is a prerequisite for modulating memory recall (Fig. 3 F-I and Fig. 6 N-Q). We found that both the direct optical stimulation of NI and the stimulation of ACC fibers in NI could increase putative excitatory cell activities (Fig 3 G-H, Fig 6 O-P and S4A-C Fig) or decrease putative inhibitory cell activities in the DG (Fig 3 I, Fig 6 Q and S4A-C Fig). These new results further highlighted the functional connectivity along

the ACC-NI-DG axis. The text and the figures were updated accordingly.

Minor concerns.

1. In Fig. 1, the authors show that optogenetic inhibition of DG SOM neurons induces fear recall in a new context (Fig. 1A & B), whereas chemogenetic inhibition prevents fear recall in the conditioned context (Fig. 1H). Does this mean that the chemogenetic inhibition overwrote the inhibition pattern of SOM neurons in association with the conditioned context? Please discuss.

The key difference between these experiments was the lack of the association with inhibition in the chemogenetic experiments. It shows that it is the very pattern of inhibitory/excitatory balance that can be associated with the memory trace, whereas indeed later chemogenetic inhibition could overwrite the inhibititory pattern created by the SOM neurons and the later chemogenetic inhibition prevented the normal restoration of the inhibitory/excitatory balance that was likely created during association. This is why we originally wrote in the discussion that: "… this kind of inhibition could overwrite the original inhibitory pattern of DG SOM cells." and "Thus, the pattern of hippocampal SOM cell inactivity needs to be temporally and spatially precise for memory recall."

2. I am also puzzled by the authors' emphasis of "a subset of" DG SOM population in memory recall. Such emphasis doesn't seem justified, since it is impossible to completely silence this cell population in its entirety, and the authors didn't test the effect of inhibiting various proportion of DG SOM neurons. In addition, the authors mentioned that "only about 55% of SOM cells were infected by the AAVs below the tip of the optic fibers" (Fig. S1G), but this figure panel did not show such data. A more representative image and group data are needed.

Indeed, it is impossible to completely silence this population of cells in its entirety (which may not be obvious to all readers, hence the emphasis), and we did not intend to silence all of them either. This is why we emphasized that we modulated only a "subset of" cell, that is not all DG SOM neurons. The exact data and measurements were not presented in the main text but only in the figure legend of Fig. S1G, where we presented all details from all mice.

3. To directly test the role of DG SOM neurons in mediating the effects of NI GABA neurons, the authors should activate NI GABA neurons and simultaneously block the GABA receptors of DG SOM neurons (or simultaneously activate these neurons). I recommend the authors to perform this experiment. At the very least, they should discuss the limitation of their current dataset.

The role of DG SOM neurons in mediating the effects of NI GABA neurons have already been demonstrated in our previous study (Szőnyi et al. 2019 Fig 1), where we showed that NI GABAergic cells innervate SOM interneurons selectively in the HIPP and their stimulation evoked strong purely GABA receptor-dependent IPSC-s on the postsynaptic SOM cells. (It is methodologically not yet possible to manipulate GABA receptors on SOM cells selectively, which would require cre-dependent intracellular inhibition of GABA receptors on dendrites. It is also not yet possible to selectively activate both HIPP SOM cells and NI GABAergic cells, which would require brain-area-dependent SOM-cre and vGAT-cre expression.)

In addition, now we investigated this specificity in the DG as well, in a series of experiments. Now, we found that that at least 87% (509/583) of virally labeled NI axonal terminals formed synapse-specific gephyrin-labeled synaptic contacts with virally labeled DG SOM cells in confocal laser scanning fluorescent immunohistochemical experiments. The few percent unidentified targets may also be SOM cells, the dendrites of which were not completely filled with viral tracer protein. This is now presented in the supplementary data section: "Extended Data for Main Fig 3".

4. The authors should provide physiological data to directly confirm that expressing eOPN3- or ArchT effectively mediates optogenetic inhibition. In addition, I am curious about the reason behind using two different optogenetic inhibition tools in a same study.

This physiological evidence has already been published and they confirmed that both eOPN3 and ArchT effectively mediates optogenetic inhibition (Mahn et al. 2021, Han et al. 2011). We used both opsins because they have different advantages. The activation of eOPN3 is fast and decays slower than ArchT (Mahn et al. 2021), which made it ideal for creating a stable inhibition of DG SOM cells (see in Fig1 A-D). eOPN3 also inhibits the axon terminals more efficiently than ArchT, therefore we used it where it was beneficial to inhibit DG SOM cell terminals as well. The activation of ArchT is also fast, but its decay time is also very fast (Han et al. 2011), thus we used this opsin where we wanted to inhibit cells in a smaller time window, e.g. CA1 SOM cells (Fig2 E-H) and DG SOM cells in aligned/shifted experiment (S2A-C Fig). The latter paper is now also cited in the manuscript.

5. AAV2/1 vectors have been used as an anterograde trans-synaptic tracing tool. Did the authors observe the labeling of DG GCs following the infection of SOM neurons with AAV2/1-hSyn-SIO-eOPN3-mScarlet virus?

We have never observed this behavior of the AAV vectors (e.g. we have never found labeled DG GCs). This is likely because we used these viruses in $7x10^{11}$ concentration, at which concentration level the possibility of anterograde trans-synaptic transfection is extremely unlikely (Zingg 2017). This is now mentioned in the Methods. Furthermore, we used CRE-dependent viruses that could not infect DG GCs, the latter of which express neither SOM nor CRE in these mice.

6. Data from the authors' group (Szőnyi et al., 2019) show that the activity of NI neurons is closely associated with locomotion. In addition, another study shows that activating NI neuromedin B neurons, which consist of mainly GABA cells, enhance animal arousal and locomotion (Lu et al. 2020). Did the authors observe any locomotor activity change following optogenetic manipulations of NI GABA neurons?

These aspects of NI cells were not investigated further in this study.

7. NI GABA neurons project to many brain areas, including the medial septum, the interpeduncular nucleus, and the median raphe, all of which are closely associated with hippocampal circuits. Stimulating NI GABA neurons or their axonal terminals will lead to the release of GABA and peptide co-transmitters in these brain areas, either directly or through antidromic activation. Therefore, some of the behavioral effects may not result from direct inhibition of hippocampal SOM neurons. The authors should discuss the caveat of their methods.

Here, we showed that direct inhibition of HIPP SOM neurons could recall associated memory traces. We have also shown that stimulation of hippocampal NI terminals could also recall them. It has never been shown that such stimulation could produce any behaviorally relevant antidromic effects at such a long distance from the soma, let alone recall memory. This is what makes the modulation of axonal arborization of remote nuclei so popular and widely accepted proof of local effect. However, even if antidromic stimulation had such an unlikely effect, it would still confirm that HIPP-projecting NI GABAergic cells can recall fear memories. It is highly unlikely, however, that this would be independent of a parallel effect on HIPP SOM cells that they selectively target and that can produce the same effect. The Method section was updated accordingly.

8. The Result section can be better organized. There are too many subsections. Some figure panels, including those related to PV neurons, could be moved to the supplementary figures.

Because of the revision, some parts of the results have now been updated and we hope that the division of the results into appropriate sub-headings will result in better clarity.

We are grateful for Reviewer 1 for the detailed and constructive review and hope that we have now improved the manuscript significantly and clarified all the necessary details.

Research Article for PLOS Biology ID: PBIOLOGY-D-22-01994R1
Title: Fear memory recall via hippocampal somatostatin interneurons

Reviewer: 2

In this manuscript, Zichó et al expand on previous findings from the same group (Szonyi et al., 2019) and investigate the effect of inhibitory networks in inducing fear memories, with special focus on SOM hilar neurons and nucleus incertus (NI) inputs. In summary, the authors show that inhibitory neurons from the hippocampus and NI contribute to reinstate patterns of activity that are important for memory expression. Non-specific manipulation of those same inhibitory networks, however, impair memory expression. This is an interesting, well executed body of work that benefits from an intelligent design and the strength of optogenetics. The paper is well written, figures are clear, information can be easily found and the amount of work is considerable. Still, the flow of the results is at times difficult to follow because little interpretation linking the results or rationale of using different experiments such as the use of different contexts, inhibition vs. activation, etc, is given. The reader gets easily lost.
In sum, I would accept the manuscript after knowing the opinion of the authors to the following major and minor questions.

We are grateful for Reviewer 2 for his/her positive opinion and the constructive and detailed comments and questions and hope that our answers and the additional series of experiments will provide further evidence for our findings.

Major:

1) In a more conceptual level, I have problems understanding the significance of the inhibition experiments, and how it translates to the more widely accepted notions of memory engrams. The introduction makes a big point that no real mechanism for precise re-activation of a given memory engram is known, but the manuscript fails to address this since reactivation of the original engram is never evaluated upon interneuron optogenetic manipulations. This would require a new battery of experiments in TRAP2 or TetTag mice that are probably too much to ask in the context of this revision. Nevertheless, this shortcoming should be properly addressed in the discussion.

The definition of engram cells is still being refined. In a strict sense, it may refer only to the episode-activated c-FOS-positive principal neurons, but more generally it can be used to describe a larger population of (excitatory/inhibitory) neurons that change their activity (become excited/inhibited) to generate a state of the network that represents a memory trace. To avoid confusion, we did not specifically talk at all about engram cell modulation in the Results section. However, our results clearly show that the fear memories we recalled are decidedly represented in a defined subpopulation of cell assemblies. This is partly because the well-defined population of cells that we manipulated are synaptically tied to their principal neurons. As a result, they had changed the excitability of a similar population of principal cells the same way, both during association and during recall. Because we modulated strictly the same cells during both association and recall, therefore these powerful inhibitory cells shaped the excitability and therefore the selection of these cells associated with the given events the same way.

Regarding why no real mechanism for precise re-activation of a given memory engram is known: entorhinal inputs alone do not seem to be suitable for memory recall because HIPP CA1 PCs and DG GCs encode information about location and context more efficiently and accurately than their entorhinal input axon terminals alone (Kitamura, T. et al., Neuron, 2015 and Cholvin et al., Neuron, 2021). This suggested that extra-cortical inputs must contribute to the refinement of the representation of these memory traces, one of which input is presented here. This is now addressed in the discussion (p 11, from line 13).

For example, in figure 1, the results of the opto-experiment are quite intriguing but reactivation of the original ensemble of engram cells in the DG is not assessed. Re-inhibition of SOM cells could lead to a precise reactivation of the original ensemble as the authors suggest, but it could also increase the mere probability of chance reactivation just by leading to the recruitment of larger ensembles of cells, as the authors actually show.

We did not "increase the mere probability of chance reactivation just by leading to the recruitment of larger ensembles of cells". This possibility was tested in control experiments, and it was ruled out. (Fig. 1 A-D "Not associated" mice). We found that disinhibition of a large ensembles of any granule cells (via SOM cells of a previously foot-shocked animal) cannot recall fear memories simply because some of these cells were involved in the encoding of a previous fear memory. In addition, we also showed that inhibition of DG SOM cells is significantly more effective for fear memory recall, if it is precisely aligned with the foot-shocks (new supplementary figure S2A-C). Therefore, disinhibition of granule cells could recall memories only if the same disinhibitory pattern had been directly associated with the original aversive stimulus (i.e. the previous foot-shocks) in a spatially and temporally precise manner.

2) In several panels (eg, Fig 2B, 3H, 4B, ...) mice show 0% freezing. This is rather unusual, and can heavily skew the results. a. Figure 2. It is weird to observe that there is basically no freezing in eOPN3 mice on the first, light-off period of the 8th day. This contrasts with the rest of experiments in the manuscript, especially with the previous experiment where the authors even discussed that "mice showed some fear behaviour because it was not possible to build a novel environment that is completely different from environment B". Can the authors explain? Could this be a general "encoding error" effect derived from the inhibition of PV neurons during shock delivery? Were the same mice tested in the environment B afterwards as in the first SOM experiment? b. Figure 3/4: Once more, the lack of freezing on the readout day, in this case in control animals, is worrying. Is there any consistent explanation for this?

Even after setting up these behavioral paradigms for all mouse strains used, freezing times between and within different transgenic mouse lines vary to a great extent. Even within the same transgenic mouse lines, individual mice always have different behavioral traits, e.g. due to their rank in the same litter, maternal behavior or small mutations. In addition, in behavioral experiments, different mice may choose different set of environmental cues that they would primarily associate with the unconditioned stimulus, which makes the behavioral response to a new environment highly variable. However, all these mouse strains are used in several experiments and all of them can learn to show significant fear recall response in both contextual and cued fear paradigms. Indeed, SOM-Cre mice always show higher, whereas PV-Cre or vGAT-Cre mice show lower freezing even after a fear conditioning protocol like in our experiments (Fig2A-D, Fig4A-D, Fig4E-G). In fact, near-zero or very low freezing times are much more likely to be expected in general under these experimental conditions.

In addition, low freezing in PV-Cre mice could not be "a general encoding error effect derived from the inhibition of PV neurons during shock delivery" because control PV-Cre mice have no light-sensitive channels yet behave the same way.

3) Figures 3 and 4 should be combined.

Part of original Fig. 3 is now combined with the original Fig. 4, whereas the original Fig. 3 is updated with new experiments.

Minor:

Fig1: The DG-SOM inhibited group ("associated" mice) does not show recall impairments in the same context (day 9), which seems at odds with similar experiments done in CA1 (Lovett-Barron et al., 2014, Szonyi et al., 2019). This needs to be explained.

This is caused by inherent differences between the CA1 and DG subnetworks because the negative effect of inhibition of DG SOM interneurons during memory acquisition is compensated by their ability to increase the size of DG memory engrams (Stefanelli et al. 2016). This is now mentioned in the Discussion section (p 11, from line 7).

Was opto-silencing done also in the same context (see my comment on chemo-opto manipulations below)?

Results from opto-silencing (re-inhibition) done also in the same (original) context shows an even stronger effect (Fig. S2C) than in a new context (Fig. S2B).

In 1A, 8th day the initial light off is indicated as a 3min period whereas in the text is stated 2min.

Thank you. We corrected it.

In 1B, was there a batch effect? Were all mice run at the same time?

No, these data are from 3 independent experiments. We merged the results from 3 cohorts of mice with similar results.

Optic fibers are placed on top of the DG, whereas SOM neurons are located mostly in the hilus. Is there any way to know to which extent the light reaches the hilus and SOM neurons are inhibited?

In these experiments, we aimed to inhibit both the soma-dendritic domains (situated in hilus) and the axon arbors (situated in molecular layer) of DG SOM cells. This is why optic fibers are placed above the molecular layer of DG, from which position both areas can be illuminated. Furthermore, we used 10mW laser intensity to generate sufficient amount of light even in the hilus. See also a light spreading calculator here: https://web.stanford.edu/group/dlab/cgi-bin/graph/chart.php

Can c-Fos be quantified within SOM cells as in exp 1G?

We did not quantify c-Fos in DG SOM cells, because they did not express c-Fos enough in the CTRL sides for comparison.

Related to this, what is the evidence that eOPN3 is actually working?

The effectiveness of eOPN3 has already been clearly demonstrated (Mahn et al. 2021). In addition, because the only difference between our control and experimental animals was their different viruses and they have shown significant behavioral differences, this further proves that eOPN3 works effectively.

I have troubles putting the opto and chemo-genetic results together, since they seem contradictory: Re-inhibiting SOM via optogenetics induces recall in a neutral context, but inhibiting SOM via chemogenetics reduces freezing in the same context. In both cases the expected effect in DG engram cells should be the same, with the difference that in the chemogenetic experiment the silencing is not specifically targeted to the "encoding" SOM cells… although they are most likely inhibited as well. Is that not contradictory? Nonetheless, to ascertain if this was an artifact derived from the method used and to highlight the specificity that authors seek it would have been interesting to inhibit the SOM neurons in the conditioned context via optogenetics in day 9 of the first experiment.

The results do not contradict, because there was a key difference between these experiments: the lack of association between the aversive events and the inhibitions in the chemogenetic experiments. It shows that it is the pattern of inhibitory/excitatory balance that can be associated with the memory trace, whereas indeed the chemogenetic inhibition overwrote the inhibititory pattern of SOM neurons and prevented the normal restoration of this inhibitory/excitatory balance created during association. This is why we originally wrote in the discussion that: "… this kind of inhibition could overwrite the original inhibitory pattern of DG

SOM cells." and "Thus, the pattern of hippocampal SOM cell inactivity needs to be temporally and spatially precise for memory recall." In addition, the results of optogenetic re-inhibition done in conditioned context as well shows an effect that is even stronger (Fig. S2C) than in a new context (Fig. S2B).

Fig. 2: Merging A-D together with the arrangement of F-G makes the figure a bit confusing. Rather than making a new figure I would consider re-arrangements between panels if the authors agree.

Yes, we moved the two sets of figure-panels further apart and thickened the line that separated them.

F. As this experiment is introduced, I understood that the goal was to test whether the memory trace of a contextual memory is lost or silent upon inhibition of CA1 SOM cells during conditioning, which was linked to decreased freezing (at least via manipulations of NI fibers) in their previous work (Szonyi paper). Re-inhibition increases freezing but this does not necessarily mean that the same memory trace has been reactivated. Once more, engram experiments would be necessary to make such claims.

Because these mice have never had similar experience before, therefore their fear memory traces must originate from these associations with these cell assemblies in a new environment where control animals show significantly less fear. These are specifically recalled by similar cell assemblies that were modulated by the optogenetic target cell population, because optical modulation affected exactly the same cells. Nevertheless, to avoid any confusion, we did not talk about engrams in the Results and we did not talk about specifically re-activating a completely identical population of engram cells. However, our results clearly showed that we recalled those specific hippocampus-dependent fear memories we created via the very same population of cells.

Is there any reason to use preferentially eOPN3 or ArchT in these different experiments?

With used both opsins because they have different advantages. The activation of eOPN3 is fast and decays slower than ArchT (Mahn et al. 2021), which made it ideal for creating a stable inhibition of DG SOM cells (see in Fig1 A-D). eOPN3 also inhibits the axon terminals more efficiently than ArchT, therefore we used it, where it was beneficial to inhibit DG SOM cell terminals as well. The activation of ArchT is also fast, but its decay time is very fast as well (Han et al. 2011), thus we used this opsin where we wanted to inhibit cells in a smaller time window, e.g. CA1 SOM cells (Fig2 E-H) and DG SOM cells in aligned/shifted experiment (Fig. S2A-C).

Fig. 3 Why 40 minutes for fos expression when in an earlier experiment 60 min were used?

The optimal time window is about 40-90 minutes, the choice of which does not affect the results. It is chosen within this window based on what was practical for the researcher.

Was it investigated whether NI GABAergic fibers also target PV cells? Although the effect of NI fibers on triggering activity in DG GCSs and recall upon re-activation is convincing, whether or not such effect is exclusively due to their action on SOM cells is not shown, and therefore the title of the section in the text is not correct. This specificity could be addressed via simultaneous manipulation of NI fibers and SOM hilar cells on the 8th day, or by showing the effect of NI fiber manipulation on SOM activation with fos/IEGs stainings. Regardless, the effect of NI fibers on PV populations should be assessed.

Although we have previously shown that NI fibers preferentially target HIPP SOM neurons (Szőnyi et al., 2019), at this Reviewer's request, here we also investigated this specifically in the DG in a series of experiments. Now, we found that that at least 87% (509/583) of virally labeled NI axonal terminals formed synapse-specific gephyrin-labeled synaptic contacts with virally labeled DG SOM cells in confocal laser scanning fluorescent immunohistochemical experiments. The few percent unidentified targets may also be SOM cells, the dendrites of which were not completely filled with viral tracer protein. This is now presented in the supplementary data section: "Extended Data for Main Fig 3".

Figure 3B would benefit from a zoomed-out view.

Now we present a zoomed-out view in Fig. S4.

Fig. 4 Minor: in the figure baseline and shock delivery occurs in the same, 6th day, whereas in the text is stated "the next day". Which of the two?

This was a typo in the text, which is now corrected.

Fig. 5
The order of the panels is again confusing. I wonder if the NI fiber and cell bodies manipulations could be merged in the same figure.

Now, we merged these figures.

Freezing levels at remote time are very high compared to the rest of the paper, which is the interpretation for this? What is the correlation of fos in the DG vs. vGAT fos in the NI at remote times?

We did not see any particular reason for this, but it was interesting indeed. We found a positive tendency of correlation between DG and NI here as well; however the number of data points were too low for drawing a statistically accurate conclusion.

5I: How is the 800% delta freezing brought about?

As it is presented in Fig 5I, change of freezing time is calculated this way: 1-(8th day freezing time/7th day freezing time). In this case: 1-(46/5,11) =-8, that is, it is 800% higher than without change. This is an outlier that we never exclude from statistics. Still the difference was significant between the groups.
There was a typo in the sign of this number that we corrected in the figure and the text.

Having the inhibition of NI cells after the recent/remote experiment and correlations seems odd, why not putting it next to the optogenetic activation of the previous figure?

These experiments are presented in the same figure because they show that NI GABAergic cells are activated during contextual fear memory recall (c-FOS), and their activity is necessary for fear memory retrieval (behavioral experiment).

If here the effect of inhibition is tested via opto- instead of chemo-genetics, why testing it in consecutive days and not in a 2-3-2 min fashion as in the rest of the paper?

This is a "lack of function" and not a "gain of function" experiment. These experiments tested whether healthy NI cells activity is a prerequisite for fear memory retrieval (we showed that they are), therefore, here we used inhibition only during contextual fear memory recall period, and therefore, we had to inhibit NI cells from the beginning of the contextual exposure.

Also, it is not clear from the text whether the effect of optogenetic inhibition was additionally tested in the different environment B. In other words, could the manipulation of NI cells that were not active during conditioning affect fear in the different context?

In these experiments, none of the NI cells are inhibited during conditioning. Therefore, there are always some cells under the optic fiber that were not active and others that were active during conditioning in the original context A. However, it cannot be tested whether the manipulation of NI cells that were not active during conditioning decrease fear in the different context, because the animals would show little fear in a different context (because it was not associated with an aversive event).

Fig. 6
This is a beautiful figure, but it doesn't add much to the story. For the amplified insets showing contacts between cortical fibers and NI INs it would be necessary to include xy projections next to the confocal images (same for fig 3B), and quantifications. Based on these grounds, this figure should be moved to supplement.

We have presented these anatomical quantifications for both Fig 3B and Fig 6 in the supplementary data file: Extended Data for Main Fig 3 and Fig6. These data were collected from confocal image stacks with xyz projections (also see in Methods page 32, from line 15).

In addition, to give further in vivo functional evidence that cortical inputs act on NI cells, we not only demonstrated that NI GABAergic neurons can directly disinhibit and reorganize firing patterns in the DG, but we also proved that cortical inputs of the NI cells from the anterior cingulate cortex can also reorganize DG firing patterns, that are the prerequisites for modulating memory recall (Fig. 3 F-I and Fig. 6 N-Q). We found that the stimulation of anterior cingulate fibers in NI could increase putative excitatory cell activities (Fig 3 G-H, Fig 6 O-P and S4A-C Fig) or decrease putative inhibitory cell activities in the DG (Fig 3 I, Fig 6 Q and S4A-C Fig). These new results further highlighted the functional connectivity along the ACC-NI-DG axis.

Discussion. I particularly enjoyed the clinical part of the discussion, but figure S9 is not visually pleasing. Personally, I think that including small bits of discussion/interpretation at the end of each results section and not only in the discussion would help navigate the manuscript, but I leave this to the discretion of the authors.

We made sure that there is an interpretation at the end of each results section.

Other minor comments:
Page 2, line 21: Han et al. 2007 did not investigate excitability.

It is corrected.

Page 5, second paragraph: The fact that these experiments were run in SOM-Cre animals is missing from the text

It is corrected.

Page 9, line 5: What environmental stimuli?

In that study we showed that NI GABAergic fibers in the HIPP CA1 region were activated by airpuff, licking, auditory tone, water, that is by different salient environmental stimuli. We have updated this text as well.

Page 10, line 3: Something cannot be "very specific". It's either specific or not.

It is corrected.

We are grateful for Reviewer 2 for her/his very detailed review and hope that we have now clarified all the necessary details and improved the manuscript significantly.

Research Article for PLOS Biology ID: PBIOLOGY-D-22-01994R1
Title: Fear memory recall via hippocampal somatostatin interneurons

Reviewer: 3

This is an interesting and impactful study, focused on control of memory encoding and recall by hippocampal somatostatin-expressing interneurons, and the NI neurons that regulate their activity. There is much to praise and little to criticize in the study. Aside from a few recommendations for improving the text and figures, I have no major concerns that would slow publication. These recommendations are described below:

We are grateful for Reviewer 3 for his/her positive opinion and constructive comments and suggestions. Please find our point-by-point responses (in blue text) below.

-Abstract Sentence 1 "Fear-related memory traces are encoded by sparse populations of hippocampal principal neurons that are recruited based on their inhibitory-excitatory balance during memory formation" On this and some other occasions the authors state fear-related memory, or simply fear memory, and the involvement of the hippocampus. It might be better to change "Fear-related memory traces" to "contextual fear memory traces" or "spatial and contextual cues of fear-related memory traces"

Because the inhibition of the elements of this pathway clearly impaired context-specific fear memory recall and hippocampal neurons are known to be responsible for contextual memory processing, originally, we called this "contextual fear memory recall". However, upon further considerations, we decided to call it simply "fear memory recall" because it is still not entirely clear whether hippocampus encodes only the context for these memories or based on the memory index theory (Goode et al. 2020) it also encodes the aversiveness or the negative emotional aspect together with the contextual information of the experience as well. In the latter case, calling it "contextual" may not be 100% justified, therefore we decided not to use it.

- Introduction, page 1, line 21 - Cai et al 2016 should also be cited here

Now we cite this paper as well.

- Introduction, page 1, line 23 - "recall.." should be changed to "consolidation and recall" Delorme et al 2021 and Raven et al 2021 should be cited

We corrected it and cited the papers suggested.

- Introduction, page 1, lines 31-33 - and also modulate principal cell reactivation during consolidation (Delorme et al, Raven et al 2021)

We corrected it and cited the papers suggested.

- Results, page 3, line 27 - Cai et al 2016 should also be cited here

Now we cite this paper as well.

- Results, page 5, lines 18-19 - this is a question to address in the discussion section, I believe, which is: What would happen if CNO were given during both training and testing?

Chemogenetic manipulations have limitations. If it was used during memory acquisition: (1) its inhibition would have lasted for several hours after the desired memory acquisition period, created association with the home cage as well, and affected the consolidation period as well. In addition, (2) chemogenetic manipulation affects much larger population of cells, the manipulations of which are not limited by the area of effective illumination intensity like in case of optical manipulations. This would have created an unphysiologically large cell activity that would probably not have been able to encode specific enough memory traces.

- Discussion, 1st 2 paragraphs - here, again, it would be nice to see some discussion of the recently-described role of SOM interneurons in fear memory consolidation (Delorme et al 2021)

At your request, we updated the discussion. See in page 10, from line 19: "SOM interneurons have an important role in the formation, consolidation and recall of memory traces across several brain regions" and we cited 4 papers including Delorme et al 2021.

- Figure 1b - Should check p-value for 1B non-associated group. There seems to be a trend that laser stimulation (SOM inhibition) during the test impairs memory? Or could this be a time-in-box effect? Possibly the only way to know is light on after a second time light off? So OFF-ON-OFF-ON?

Because of the time-in-box effect, CTRL mice accommodated to the box and by the time we switched on the light they showed less fear behavior. The same is true for the Non-associated group as well during the light ON period. Statistically, however, there was no difference between CTRL and Non-associated mice during light ON period (Mann-Whitney U-test, p value=0.749). All other statistical details are presented in the Supplementary text (Extended Data for Fig1).

- Figures 2 and 5 - it would be nice if these panels were laid out differently, as presented it is hard to follow - i.e., having experimental data described later in the text presented above data described earlier

Now, we moved the two sets of figure-panels further apart and thickened the line that separated them in both Fig 2 and 5, however we had to make some sacrifices to achieve a more efficient use of space in illustrations.

We are grateful for Reviewer 3 for her/his review and hope that we have now clarified all the necessary details and improved the manuscript significantly.
