# Supplementary material for: Fear memory recall involves hippocampal somatostatin interneurons
Source: PLoS Biol. 2023 Jun 8;21(6):e3002154. doi: 10.1371/journal.pbio.3002154 (PMC10284381; doi:10.1371/journal.pbio.3002154)
Supplement: Attachment — Submitted filename: Zicho_Response to Reviewers.pdf [file pbio.3002154.s022.pdf]

Response to comments by the Reviewers

Research Article for PLOS Biology ID: PBIOLOGY-D-22-01994R2
Title: Fear memory recall involves hippocampal somatostatin interneurons

We thank the reviewers for their positive assessment of our work. Below we present the Reviewers' unchanged comments in full (in black text), together with our responses (in blue text).

Reviewer #1

I appreciate the authors' efforts of addressing my concerns. They have provided additional experimental evidence to support the conclusion that hippocampal SOM neurons and NI GABA neurons participate in memory formation and recall. More specifically, they now show that aligning optogenetic stimulation with aversive stimuli produces more effective association (Fig. S2A-C). They ephys recordings in vivo now more convincingly demonstrate the functional connectivity of the ACC-NI-DG pathway (Figs. 3F-I, 6N-Q, and S4).

The revisions in Discussion are also appropriate.

I only have one minor concern. The retrograde tracing data shown in Fig. S3C illustrate only a small number of labeled neurons in the NI (one or two cells per section). It will be helpful if the authors could clarify.
We have explained this in the corresponding Results section (page 6) "rabies tracing is less efficient between distant brain regions". Additionally, there are some cell type specific differences in labeling efficiency (Sun et al. 2019 Mol. Neurodeg.).

Overall, the manuscript has been substantially strengthened by the revisions. I am happy to support its publication at Plos Biology.

We thank the reviewer for his support of our work.

Reviewer #2

I thank the authors for their detailed responses and new additions to the manuscript. In vivo electrophysiology complements well the results on NI to DG SOM connectivity.

I agree with some of the responses from the authors while finding others no entirely convincing. Specifically, I would like to make the following final comments:I believe the align vs. shift experiment is interesting and well-conceived. Unfortunately, the increase in freezing in B, although significant, is difficult to interpret biologically (from 2.6 to 6.9 %) and falls far from the bigger effect observed in the first experiment, in which additionally a different opsin had been used. These discrepancies should be explained or at least justified in the text.
Indeed, it was because of a different opsin used. The figure legend of S2B Fig. is now updated to explain this: "These ArchT mediated recalls in environment B are significant but less effective than those mediated by eOPN3 that had a longer deactivation time during fear conditioning (Fig 1)."

I completely agree with the response of the authors to my question about "increasing chance reactivation alone".
Indeed, the non-associated mice are the perfect control for this, which I overlooked. Stressing this point, maybe when talking about figure 1D, could support the text.
"Non-associated" experiments are emphasized in Results (page 4): "However, it also shows that a nonspecific inhibition of SOM cells, even after a powerful, recent fearful event, cannot recall memory unless the same subset of SOM cells primes their target GCs during memory acquisition." And also emphasized in the Discussion (page 10): "This was specific because if the experimental inhibition of DG SOM cells was not associated with a fearful event, then their re-inhibition could not recall any fear memories in a novel environment."

Evidence that the optogenetic approaches effectively inhibit target cells is still missing, even though it was asked by me and reviewer 1. Previous works demonstrating its effectiveness supports its choice, but methodological differences might lead to different results. This requires further attention.
Besides the previously demonstrated effectiveness of eOPN3 (Mahn et al. 2021) in several other cell types (cortical pyramidal cells, nigro-striatal dopaminergic cells, thalamo-cortical neurons) and besides the significant behavioral effects of eOPN3 in our experiments, the effective inhibitory effect of eOPN3 in our system was also supported by the fact that it always had an effect opposite to excitatory opsins in all of our experiments.

It would be desirable to adopt a common formatting for the graphs and data depiction (e.g., showing or not individual data points in the bar graphs). Additionally, I and other reviewers commented on the strange arrangement of figures (such as fig2 and 5), but these were disregarded. I leave this to the discretion of the authors or the editors.
As per PLOS Data Policy, now, we have provided the individual numerical values that underlie the graphs' data displayed in all figure panels, regardless of their representations in the figures. Additionally, the layout of the figures has been designed to be as concise as possible, which can be changed on request during proof editing if necessary.

Regarding my question about differences in the effect of SOM-inhibition in CA1 and DG at recall, I find the answer of the authors to be a bit superficial ("caused by inherent differences between CA1 and DG subnetworks") and inaccurate (SOM inhibition in CA1 would probably also increase activity in this area). Anyway, the conclusion for the manuscript does not change.
Indeed, it was not explained in details because it is still an intensively researched topic. Any current explanation would have required speculation and a comprehensive review of the current literature, which in our opinion would have been beyond the scope of this article.

More generally, and concerning the response letter, I would advise strongly against overinterpreting beyond what is strictly shown by the data. The experiments do show the effect of re-inhibition in memory encoding and recall, but in their response the authors state: "This is partly because the well-defined population of cells that we manipulated are synaptically tied to their principal neurons. As a result, they had changed the excitability of a similar population of principal cells the same way, both during association and during recall. Because we modulated strictly the same cells during both association and recall, therefore these powerful inhibitory cells shaped the excitability and therefore the selection of these cells associated with the given events the same way". While I agree with the feasibility of this interpretation, strictly speaking, this was never shown in the present study. Once more, engram studies and/or slice physiology of principal cells during optogenetic inhibition of SOM cells would be necessary for this (which is probably out of the scope of the present manuscript, and not needed). Something similar occurs when the authors repeatedly refer to "patterns of activity" that are lost or reinstated after chemo- or opto-inhibition, respectively, because such "patterns" were never measured and are hardly reproducible with opto-protocols. The results are consistent and solid, but interpretation can go in different directions and authors should tone down some of their statements.
We agree with the Reviewer that over-interpretation of the data should be avoided. In our response letter, we have only used the expression of neuronal "activity pattern" in a general sense. Indeed, correlation of the "activity pattern" of the neurons in this study and exact engram pattern may be addressed in more details in future research.

We are also grateful for this Reviewer for her/his constructive comments and support of our work.

Reviewer #3

The authors have done an outstanding job of responding to prior concerns. The present study will shape our thinking in the field for years to come, and they should be congratulated on this nice work.

We are grateful for this Reviewer as well for her/his review and support of our work.
